# Mercury, Cadmium, and Lead Levels in Human Placenta: A Systematic Review

**DOI:** 10.1289/ehp.1204952

**Published:** 2012-05-16

**Authors:** María D. Esteban-Vasallo, Nuria Aragonés, Marina Pollan, Gonzalo López-Abente, Beatriz Perez-Gomez

**Affiliations:** 1 Subdirectorate for Health Promotion and Prevention, Madrid Regional Health Authority, Madrid, Spain; 2 National Center for Epidemiology, Carlos III Institute of Health, Madrid, Spain; 3 Consortium for Biomedical Research in Epidemiology & Public Health (CIBER en epidemiología y Salud Pública-CIBERESP), Madrid, Spain

**Keywords:** biomonitoring, cadmium, lead, mercury, metals, placenta

## Abstract

Background: Placental tissue may furnish information on the exposure of both mother and fetus. Mercury (Hg), cadmium (Cd), and lead (Pb) are toxicants of interest in pregnancy because they are associated with alterations in child development.

Objectives: The aim of this study was to summarize the available information regarding total Hg, Cd, and Pb levels in human placenta and possible related factors.

Methods: We performed a systematic search of PubMed/MEDLINE, EMBASE, Lilacs, OSH, and Web of Science for original papers on total Hg, Cd, or Pb levels in human placenta that were published in English or Spanish (1976–2011). Data on study design, population characteristics, collection and analysis of placenta specimens, and main results were extracted using a standardized form.

Results: We found a total of 79 papers (73 different studies). Hg, Cd, and Pb levels were reported in 24, 46, and 46 studies, respectively. Most studies included small convenience samples of healthy pregnant women. Studies were heterogeneous regarding populations selected, processing of specimens, and presentation of results. Hg concentrations > 50 ng/g were found in China (Shanghai), Japan, and the Faroe Islands. Cd levels ranged from 1.2 ng/g to 53 ng/g and were highest in the United States, Japan, and Eastern Europe. Pb showed the greatest variability, with levels ranging from 1.18 ng/g in China (Shanghai) to 500 ng/g in a polluted area of Poland.

Conclusion: The use of the placenta as a biomarker to assess heavy metals exposure is not properly developed because of heterogeneity among the studies. International standardized protocols are needed to enhance comparability and increase the usefulness of this promising tissue in biomonitoring studies.

During gestation, potentially harmful pollutants circulating in the blood of pregnant women can reach the fetus, although possible mother-to-child transmission of these toxic substances is modulated by the placenta. Some authors (Iyengar and Rapp 2001a; Subramanian and Iyengar 1997) have proposed the use of placental tissue as a noninvasive exposure biomarker for different organic and inorganic pollutants. This tissue, usually discarded after birth, is easy to obtain and may furnish information on the exposure of both mother and fetus.

Among the contaminants eligible for being studied in this tissue, some heavy metals are of special interest when it comes to gestation. Exposure to either mercury (Hg) or lead (Pb) has well known deleterious effects on the intellectual development of children (Jakubowski 2006), and embryotoxic and fetotoxic effects associated with exposure to cadmium (Cd) have also been described (Hazelhoff et al. 1987).

We reviewed published research on total Hg, Cd, and Pb levels in human placenta, both in pregnant women from the general population and in those with specific exposures, as well as the factors related to such levels, in order to summarize available information for researchers considering the use of placenta to biomonitor exposure to these metals.

## Methods

*Search strategy*. The systematic search and review processes were conducted in accordance with the Preferred Reporting Items for Systematic Reviews and Meta-Analyses (PRISMA) Statement criteria (Liberati et al. 2009). We searched PubMed/MEDLINE (http://www.ncbi.nlm.nih.gov/pubmed/), EMBASE (http://www.embase.com/home), LILACS (http://lilacs.bvsalud.org/en/), OSH (http://www.oshupdate.com/), and Web of Science (http://www.accesowok.fecyt.es) databases using the following MeSH terms: “placenta” successively combined with “pollutants,” “cadmium,” “mercury,” “lead,” and “biomonitoring.” This was supplemented by manual searches based on the references cited in the papers initially identified. We considered only studies on human subjects published in English or Spanish from January 1976 through November 2011. This strategy yielded 1,629 references [see Supplemental Material, Figure S1 (http://dx.doi.org/10.1289/ehp.1204952)]. From these, we selected the studies that furnished original quantitative data on total Hg, Cd, or Pb levels measured in human placental tissue samples.

*Data extraction*. Using a standard, purpose-designed form, we extracted the following data from each paper: *a*) study design, date and place, participants, sampling method and size, inclusion and exclusion criteria, and request for informed consent; *b*) protocol for collection, storage, processing, and analysis of biologic specimens; and, *c*) results, including metal levels and related factors. Where concentrations were expressed in dry weight (dw), we calculated wet weight (ww) by dividing the figure by the dw/ww ratio, if provided, or by a value of 6, if the dw/ww ratio was not provided (Iyengar and Rapp 2001b); where the type of weight was unspecified, we allocated ww or dw in light of the range of values used. We report the arithmetic and/or geometric mean, and we include SD, median, and range where provided. In view of the heterogeneous results displayed by the original studies, we felt that quantitative pooling was inappropriate.

## Results

### Description of the Studies

We included a total of 79 reports on 73 different studies. Twenty-four studies reported placental Hg levels, 46 reported Cd levels, and 46 reported Pb levels; papers frequently provided results on more than one trace metal. The main characteristics of each study are described in Supplemental Material, Table S1 (http://dx.doi.org/10.1289/ehp.1204952), and metal levels are summarized in Supplemental Material, Tables S2–S4. The full text was unavailable for 8 papers; for those papers, data shown in the summary tables came from the abstracts or from reviews of those studies. The number of papers increased over time, reaching a peak in the past decade because of studies addressing placental Cd and Pb (see Supplemental Material, Figure S2). Whereas older studies were mainly undertaken in the United States and Western Europe, studies targeting populations in India, China, Eastern Europe, and South America began to be published in the 1990s, and accounted for the bulk of published works from 2000 and later.

*Design: designated objectives and study subjects.* Most of the papers reviewed were cross-sectional studies with three distinct types of objective, often combined in the same report. Most studies adopted a biomonitoring standpoint. Among these, some included variability by region of origin, rurality, or lifestyle aspects, with smoking being the determinant most frequently studied, especially in studies measuring Cd. Another objective was the acquisition of in-depth knowledge on the metabolism of metals and their passage through the placenta, assessing the placenta’s possible barrier effect by examining the link between placental levels and other related biomarkers. Indeed, 25 studies collected other complementary biologic specimens, such as maternal and umbilical cord blood, cord tissue, maternal milk, maternal or newborn hair and urine, or meconium. Finally, some researchers assessed the association between metal levels and disorders in the progress of pregnancy or newborn development, including premature membrane rupture or preterm births (Ahamed et al. 2009; Baghurst et al. 1991; Falcón et al. 2003a); gestational age and anthropometric data (Ahamed et al. 2009; Baghurst et al. 1991; Falcón et al. 2003a, 2003b; Frery et al. 1993; Guo et al. 2010; Kantola et al. 2000; Khera et al. 1980; Kippler et al. 2010; Klapec et al. 2008; Llanos and Ronco 2009; Loiacono et al. 1992; Osada et al. 2002; Pitkin et al. 1976; Roels et al. 1978; Ronco et al. 2005a; Saxena et al. 1994; Stasenko et al. 2010; Tekin et al. 2011a, 2011b; Wibberley et al. 1977; Yang et al. 1997; Zhang et al. 2004); oligohydramnios (Terrones-Saldivar et al. 2008); intrauterine growth restriction (IUGR) (Klapec et al. 2008; Llanos and Ronco 2009; Osada et al. 2002); Apgar score at birth (Zhang et al. 2004); and fetal malformations or stillbirths (Baghurst et al. 1991; Khera et al. 1980; Saxena et al. 1994; Scaal et al. 1998; Wibberley et al. 1977).

Most studies included small groups of women, and only 19 included > 100 participants. Just 2 papers provided participation rates (Al Saleh et al. 2011; Lagerkvist et al. 1996), which was > 90% in both cases, and another study specified the dropout rate after recruitment (Gundacker et al. 2010). The inclusion criteria were frequently poorly described, and in some cases were limited to a mere reference to geographic origin (Baranowska 1995; Karp and Robertson 1977; Schramel et al. 1988). The most common practice of the reviewed studies was to include nonrandomly selected convenience samples of healthy pregnant women without known risk exposures and who had full-term births and normal neonates. However, some studies included pregnant women with a higher probability of environmental exposure to heavy metals, whether by occupational exposure (Berlin et al. 1992; Khera et al. 1980; Mayer-Popken et al. 1986; Yang et al. 1997) or high environmental exposure (Baghurst et al. 1991; Diaz-Barriga et al. 1995; Fahim et al. 1976; Guo et al. 2010; Lagerkvist et al. 1996; Loiacono et al. 1992). Other studies targeted women who experienced problems with fetal development or pregnancy (Baghurst et al. 1991; Khera et al. 1980; Saxena et al. 1994; Scaal et al. 1998; Terrones-Saldivar et al. 2008; Wibberley et al. 1977). Some studies used type of delivery as an inclusion criterion, although only 15 reports provided this information. Of these, 9 covered only noncomplicated vaginal births (Ahamed et al. 2009; Capelli et al. 1986; Frery et al. 1993; Kantola et al. 2000; Needham et al. 2011; Pitkin et al. 1976; Stasenko et al. 2010; Tsuchiya et al. 1984; Zagrodzki et al. 2003), 4 combined vaginal and cesarean (C)-section births (Bush et al. 2000; Sorkun et al. 2007; Tekin et al. 2011a, 2011b; Terrones-Saldivar et al. 2008), and 1 exclusively included placentas from C-section births (Fagher et al. 1993). Regarding ethical considerations, 28 papers expressly referred to informed consent as a prerequisite for study participation and 14—mostly recent—mentioned the approval of an ethics committee.

*Analytical methodology: collection, processing, and analysis of biologic specimens.* Pretreatment of the placenta, which was not always specified, varied widely. In general, the organ was frozen and stored, either whole, processed, or in the form of the specimen collected. Usually, the placenta had an initial washing to remove blood or clots; in some studies the decidua basalis, chorionic plate, or connective tissue and blood vessels were eliminated, whereas in others only the cotyledon was analyzed. In most instances, a single spot specimen of placenta weighing only a few grams was obtained, without specifying the placental area sampled. Other researchers collected several specimens from different areas, typically to analyze them jointly and sometimes to carry out intercomparisons among the individual results (Kippler et al. 2010; Lagerkvist et al. 1996; Piasek et al. 2001). Finally, in other studies the complete placenta or one of its halves was homogenized, and part of this preparation was analyzed.

In general, different types of spectrometry were used, except in one case in which both Cd and Pb were determined using a hanging Hg drop electrode (Kutlu et al. 2006) and in five studies in which Hg was measured using an unspecified Hg analyzer (Hsu et al. 2007) or instrumental neutron activation analysis (Grant et al. 2010; Horvat et al. 1988; Llanos and Ronco 2009; Ward et al. 1987). These details were not always provided (Karp and Robertson 1977).

Information on laboratory quality controls differed between the earliest and the more recent papers: Before the year 2000, only 11 studies (26.8%) specified having internal quality controls and 6 studies (14.6%) mentioned external controls. In contrast, all studies published after 2000 except 1 (Terrones-Saldivar et al. 2008) reported the use of standardized quality control procedures that generally comprised evaluation of accuracy and precision by analysis of certified reference material, and 2 studies included additional interlaboratory comparisons (Singh et al. 2010; Zagrodzki et al. 2003). Only half of the studies specified the limit of detection (LOD), merely stating the value or including the procedure used to treat values below it.

In the reviewed studies, there was no uniform pattern for reporting results: 28 studies described levels in terms of wet weight, 26 in terms of dry weight, and the remainder did not specify this aspect. Four papers used their own data to calculate the dw/ww ratio, which was 6.32 (Capelli et al. 1986), 6.2 (Schramel et al. 1988), 6.9 ± 1.3 (mean ± SD; Osada et al. 2002) and 5.7 (range, 4.8–7.7) (Kippler et al. 2010), respectively, whereas 2 papers used a fixed ratio of 6.0 (Baghurst et al. 1991; Frery et al. 1993). As units of measurement, most authors used nanograms or micrograms per unit weight, although nanomoles and parts per billion were also employed; some reports seemed to contain errors in the units (Kantola et al. 2000; Li et al. 2000; Loiacono et al. 1992; Singh et al. 2010).

*Statistical analysis and reporting of results.* Most of the studies (84.7%) provided arithmetic means, usually accompanied by SDs. Other widely used indicators were median (29 studies) and range (28 studies); geometric mean was reported in 7 papers. With one exception (Al Saleh et al. 2011), authors did not include confidence intervals nor did they mention the evaluation of outliers or the use of robust measures of central trend.

Studies assessing associations with risk factors generally furnished correlation coefficients drawn from univariate analyses, or differences in means derived from stratified analyses; few reports carried out multivariate analyses (Al Saleh et al. 2011; Falcón et al. 2002, 2003a, 2003b; Gundacker et al. 2010; Kantola et al. 2000; Kippler et al. 2010; Klapec et al. 2008; Lagerkvist et al. 1996; Loiacono et al. 1992; Osada et al. 2002; Zhang et al. 2004).

### Placental Trace-Metal Levels

The individual results yielded by studies on Hg, Cd, and Pb that we reviewed are presented in Supplemental Material, Tables S2, S3, and S4, respectively (http://dx.doi.org/10.1289/ehp.1204952).

**Figure 1:**
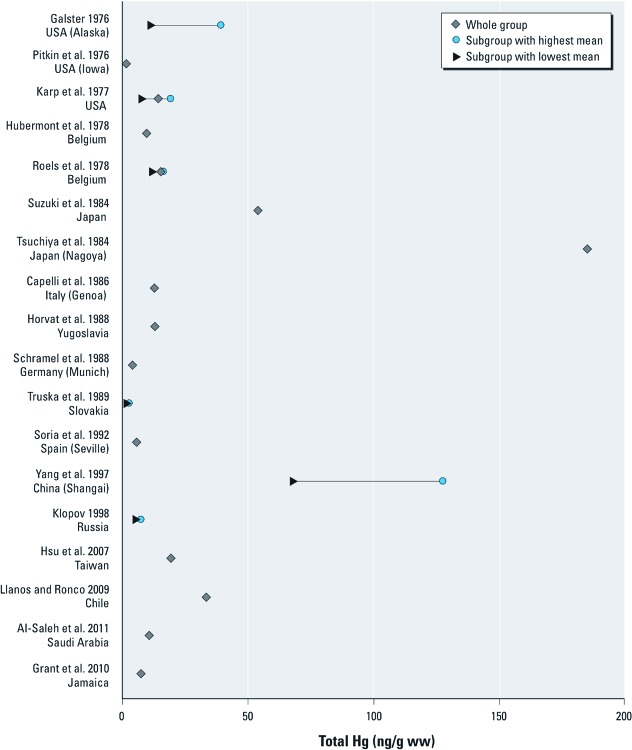
Total Hg levels [arithmetic mean (ng/g ww)] in human placenta (1976–2011).

*Total Hg.* Twenty-four studies reported total Hg levels, although only 4 included > 100 women [see Supplemental Material, Table S2 (http://dx.doi.org/10.1289/ehp.1204952)]. The arithmetic mean Hg levels reported in those studies having this information are presented in Figure 1. Hg concentrations varied widely; the highest were found in China (Shanghai) and Japan (Suzuki et al. 1984; Tsuchiya et al. 1984; Yang et al. 1997), where the mean exceeded 50 ng/g, and in the Faroe Islands, with a median of approximately 90 ng/g (Needham et al. 2011). In contrast, in Germany (Schramel et al. 1988) and the Ukraine (Zadorozhnaja et al. 2000), most samples were < LOD. Three studies also provided the proportion of organic to inorganic Hg (Capelli et al. 1986; Soria et al. 1992; Suzuki et al. 1984).

Relationship between Hg in the placenta and other specimens. Studies that compared Hg concentration in the placenta with levels in maternal or cord blood yielded conflicting results. In general, higher Hg levels were observed in the placenta (Galster 1976; Gundacker et al. 2010; Hsu et al. 2007; Pitkin et al. 1976; Tsuchiya et al. 1984; Yang et al. 1997), suggesting an accumulation of this metal during pregnancy. Nevertheless, other authors reported placental levels that were lower than blood levels (Hubermont et al. 1978; Roels et al. 1978; Truska et al. 1989).

Almost all studies reported a positive correlation between placental Hg and venous (Al Saleh et al. 2011; Galster 1976; Marques et al. 2007; Needham et al. 2011; Tsuchiya et al. 1984) or arterial cord blood (Soria et al. 1992), umbilical tissue (Needham et al. 2011), or maternal and neonate hair Hg (Marques et al. 2007). For maternal blood, however, results were inconsistent (Al Saleh et al. 2011; Roels et al. 1978).

Association between Hg, maternal characteristics, and exposure to possible risk factors. In general, no association was found with maternal age or parity (Pitkin et al. 1976; Roels et al. 1978), although Al Saleh et al. (2011) described a significant association with body mass index (BMI) after controlling for possible confounders.

Fish intake, the most common source of population exposure to Hg, was the risk factor that was most widely studied for Hg levels. The highest levels were found in regions with high fish consumption, such as Japan and the Faroe Islands (Needham et al. 2011; Tsuchiya et al. 1984). In a small study on Eskimo women, Galster (1976) also observed a positive association between diets very rich in fish or whale meat and placental Hg. However, in two studies (Hsu et al. 2007; Marques et al. 2007), differences in intake during pregnancy were not associated with Hg levels in populations whose diet included lower fish consumption, although an association with consumption prior to pregnancy was suggested (Hsu et al. 2007).

Few studies have examined the role of nondietary exposures to Hg. Some researchers have reported an absence of association with drinking habits (Roels et al. 1978), dental fillings (Hsu et al. 2007), or tobacco abuse (Roels et al. 1978). Some studies reported contradictory results related to residence in areas with industrial exposure (Karp and Robertson 1977; Zadorozhnaja et al. 2000), whereas others found no differences between women living in rural and urban settings (Roels et al. 1978; Truska et al. 1989). Data on occupational exposures were equally sparse: One small study of Chinese women exposed to Hg at a lamp factory reported elevated placental levels (Yang et al. 1997), yet we found no studies that had measured placental Hg levels among other occupationally exposed women, such as dentists or chloralkali plant workers.

Possible effects of Hg on the placenta or pregnancy. Karp and Robertson (1977) described a possible inhibitory effect of Hg on some placental enzymes implicated in the metabolism of carbohydrates and steroids; however, no studies reported an association between total placental Hg and the anthropometric variables of neonates (Gundacker et al. 2010; Pitkin et al. 1976; Roels et al. 1978) or gestational age (Roels et al. 1978). A similar result was found in a study of fetuses with malformations of unknown origin, in which Scaal et al. (1998) failed to observe evidence of Hg implication.

**Figure 2:**
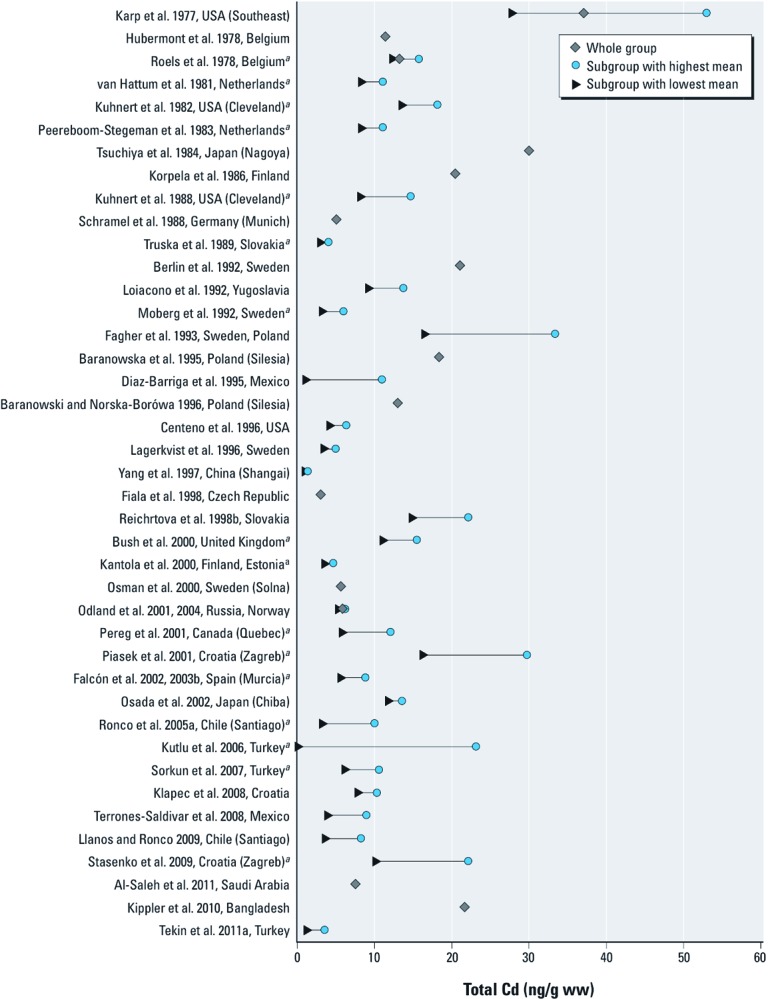
Cd levels [arithmetic mean (ng/g ww)] in human placenta (1976–2011). ^a^Studies comparing smokers to nonsmokers.

*Cd.* We located 46 studies reporting placental levels of Cd; 17 of these had > 100 participants [see Supplemental Material, Table S3 (http://dx.doi.org/10.1289/ehp.1204952)]. The average level of Cd varied widely (Figure 2)—ranging from 1.2 ng/g in Shanghai (Yang et al. 1997) to 53 ng/g in an urban area of the United States (Karp and Robertson 1977)—and was particularly high, with values >20 ng/g, in some groups of pregnant women from Japan (Tsuchiya et al. 1984), Bangladesh (Kippler et al. 2010), Turkey (Kutlu et al. 2006), and Eastern Europe (Fagher et al. 1993; Piasek et al. 2001; Reichrtova et al. 1998b; Stasenko et al. 2010).

Comparative analysis of Cd in placenta and other specimens. Cd concentrations in the placenta were much higher than those found in maternal and cord blood in all studies (Hubermont et al. 1978; Korpela et al. 1986; Kuhnert et al. 1982; Lagerkvist et al. 1996; Needham et al. 2011; Roels et al. 1978; Tsuchiya et al. 1984) except one (Truska et al. 1989). Placental Cd concentrations were 10 times higher than that detected in maternal blood in one study (Roels et al. 1978) and up to 100 times higher than that of cord blood in another (Needham et al. 2011). These data, along with the difference in placental Cd levels between the first trimester and at term (Kantola et al. 2000), indicate that Cd accumulates in the placenta during gestation and support the view that this organ is an efficient (Baranowska 1995; Kuhnert et al. 1982; Roels et al. 1978; Schramel et al. 1988) or partially efficient barrier (Korpela et al. 1986; Needham et al. 2011) against this metal.

The Cd concentration in placenta was reported to be positively correlated with maternal blood Cd (Al Saleh et al. 2011; Lagerkvist et al. 1996; Odland et al. 2001; Roels et al. 1978; Schramel et al. 1988; Zhang et al. 2004)—both in plasma and in erythrocytes (Truska et al. 1989)—and with maternal urinary Cd (Kippler et al. 2010). The relationship between placental and cord blood levels was somewhat less clear; although some studies found a correlation between them (Kippler et al. 2010; Needham et al. 2011; Tsuchiya et al. 1984; Zhang et al. 2004), others failed to do so (Al Saleh et al. 2011; Kuhnert et al. 1982; Schramel et al. 1988). No association with Cd levels in breast milk was observed (Schramel et al. 1988), although few studies assessed this association.

Effects of collection, processing, and preservation of specimens. Pretreatment of placentas may be associated with higher levels of Cd in the specimen (Lagerkvist et al. 1996). In terms of the metal’s distribution in this organ, available data were contradictory: Some studies suggested that Cd was uniformly distributed throughout the placenta (Lagerkvist et al. 1996; Piasek et al. 2001), and others described differences between samples from the central and peripheral areas, without any clear gradient (Kippler et al. 2010). In placentas from smokers, the Cd concentration seemed to be higher on the maternal side (Zhang et al. 2004). Higher levels of Cd were also observed in placentas with parenchymal calcifications, regardless of smoking habit, gestational age, or birth weight (Frery et al. 1993). Milnerowicz (1993) reported that Cd levels were lower in fetal membranes.

Association between maternal characteristics and exposure to possible risk factors. The efficiency of the placenta as a barrier against the passage of Cd might be modulated by maternal polymorphisms in genes that code for metal-binding metallothionein protein (Tekin et al. 2011a). Most studies found no association between placental Cd and parity or maternal age (Frery et al. 1993; Kantola et al. 2000; Klapec et al. 2008; Loiacono et al. 1992; Roels et al. 1978; Terrones-Saldivar et al. 2008; Zadorozhnaja et al. 2000), although in some cases higher levels were detected in older mothers (Al Saleh et al. 2011; Fiala et al. 1998). In another U.S. study, a higher Cd concentration was observed in multiparous women, albeit exclusively among smokers (Kuhnert et al. 1988).

Among environmental exposures, smoking was the most widely studied factor. Published results clearly show that placental Cd levels in pregnant smokers are two to three times higher than those in pregnant nonsmokers (Bush et al. 2000; Falcón et al. 2002, 2003b; Kantola et al. 2000; Kuhnert et al. 1982; Kutlu et al. 2006; Lagerkvist et al. 1996; Moberg et al. 1992; Osman et al. 2000; Peereboom-Stegeman et al. 1983; Pereg et al. 2001; Piasek et al. 2001; Roels et al. 1978; Ronco et al. 2005a, 2005b; Sorkun et al. 2007; Stasenko et al. 2010; Zhang et al. 2004). Several authors reported a positive correlation with the number of cigarettes smoked daily (Bush et al. 2000; Falcón et al. 2002; Pereg et al. 2001); however, in a small study in the Netherlands, very heavy smokers (20 to 60 cigarettes/day) had lower levels than did intermediate smokers, suggesting that a limited amount of Cd could be retained in the placenta (Peereboom-Stegeman et al. 1983). Furthermore, some studies found no evidence of any association between tobacco use and placental levels of Cd (Fagher et al. 1993; Frery et al. 1993; Odland et al. 2001; Terrones-Saldivar et al. 2008). Only one Turkish study specifically reported Cd levels in women with passive tobacco exposure, and levels were higher in those women compared with nonsmokers (Kutlu et al. 2006).

Important differences in placental Cd levels have been observed among pregnant nonsmokers by geographic region of origin (Kantola et al. 2000; Odland et al. 2004), indicating that these levels also reflect variations in other environmental exposures. Several studies in residents in heavily polluted areas (Baranowska 1995; Zhang et al. 2004) or in the vicinity of metal installations, such as Pb smelters (Lagerkvist et al. 1996; Loiacono et al. 1992; Tabacova et al. 1994) or iron mines (Reichrtova et al. 1998b), generally registered high levels of Cd. Urban pollution also seemed to increase placental Cd (Baranowska 1995; Fagher et al. 1993; Falcón et al. 2002; Reichrtova et al. 1998b; Sorkun et al. 2007), even when smoking was taken into account (Fagher et al. 1993; Guo et al. 2010; Roels et al. 1978; Sorkun et al. 2007). In addition, a recent study in China suggested an association with indoor pollution (Guo et al. 2010). The few studies of occupational settings did not show a clear relationship with Cd levels (Berlin et al. 1992; Roels et al. 1978; Yang et al. 1997).

In terms of diet, one study reported lower placental Cd in women with high iron levels and low intake of cereals at the end of pregnancy (Moberg et al. 1992); no association was found with drinking habits (Roels et al. 1978).

Possible effects of Cd on the placenta or pregnancy. Because Cd accumulates in the placenta, higher levels would be expected in pregnancies of longer duration. Nonetheless, the studies we reviewed either found no association with gestational age (Frery et al. 1993; Loiacono et al. 1992; Roels et al. 1978) or reported an inverse relationship (Falcón et al. 2003b; Kippler et al. 2010). One small study observed higher Cd levels in preterm compared with full-term C-section placentas, although this difference was not statistically significant (Fagher et al. 1993). However, Zhang et al. (2004) found no association with prematurity. In regard to other gestational problems, Terrones-Saldivar et al. (2008) observed higher levels in placentas from pregnancies with severe oligohydramnios.

Research in this field has also assessed fetal development and neonatal anthropometric data. Three small studies reported higher Cd in placentas from pregnancies with IUGR (Klapec et al. 2008; Llanos and Ronco 2009; Osada et al. 2002), although the differences were not always statistically significant (Osada et al. 2002). Other studies described a negative correlation with birth weight (Kippler et al. 2010; Ronco et al. 2005a; Stasenko et al. 2010), neonatal length (Stasenko et al. 2010), and chest circumference (Kippler et al. 2010). However, a few studies failed to find any association between Cd and infant measures (Guo et al. 2010; Tekin et al. 2011a) or neonatal Apgar score (Zhang et al. 2004).

In terms of placental metabolism, the aspect most commonly described was the direct link between placental Cd and high levels of placental metallothionein (Kippler et al. 2010; Ronco et al. 2006; Sorkun et al. 2007; Tekin et al. 2011a). Although this metalloprotein is considered a protective cellular mechanism against Cd, its increase might, in turn, affect the passage of zinc into the fetus (Kippler et al. 2010; Tekin et al. 2011a). Other reported effects of Cd include a decrease in placental synthesis of leptin (Stasenko et al. 2010) and increased activity of enzymes implicated in the metabolism of carnitine and carbohydrates (Karp and Robertson 1977). Pereg et al. (2001) found no association between Cd and placental bulky DNA adduct levels.

**Figure 3:**
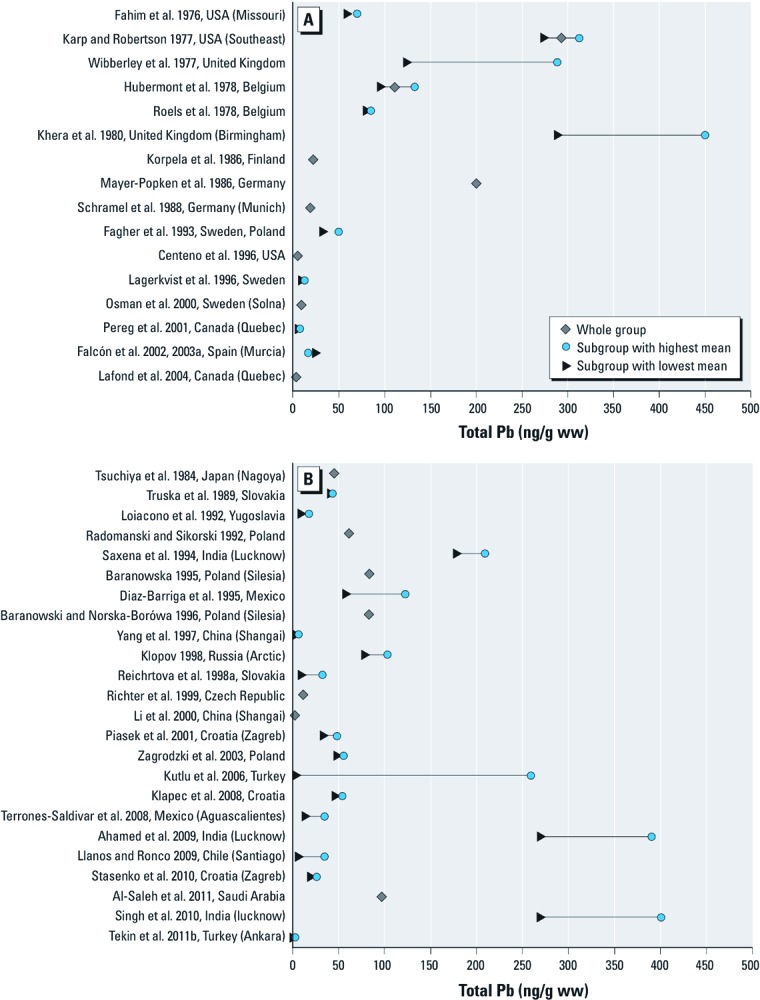
Pb levels [arithmetic mean (ng/g ww)] in human placenta (1976–2011). Studies undertaken in (*A*) Western Europe and North America and (*B*) Eastern Europe, Latin America, and Asia.

*Pb.* Placental Pb concentrations were reported in 46 studies, 15 of which had > 100 participants [see Supplemental Material, Table S4 (http://dx.doi.org/10.1289/ehp.1204952)]. Average Pb levels ranged from 500 ng/g in women residing in Silesia, a heavily polluted region of Poland (Baranowska 1995), to 1.18 ng/g in Shanghai (China) (Li et al. 2000). Figure 3 presents the reported arithmetic means of placental Pb. Although concentrations > 100 ng/g were commonly observed in Western Europe and North America in the 1970s, since the 1980s Pb levels have remained < 50 ng/g. Some studies conducted during the last 20 years in Eastern Europe, South America, Asia, and North Africa still reflect very high levels of placental Pb, particularly in India (Ahamed et al. 2009; Saxena et al. 1994; Singh et al. 2010).

Comparative analysis of Pb in the placenta and other specimens. Placental Pb levels were usually lower than those found in maternal (Baghurst et al. 1991; Baranowska 1995; Lagerkvist et al. 1996) or cord blood (Baghurst et al. 1991; Baranowska 1995; Hubermont et al. 1978; Korpela et al. 1986; Lagerkvist et al. 1996; Mayer-Popken et al. 1986), both in plasma and erythrocytes (Truska et al. 1989). However, the opposite has also been reported (Hubermont et al. 1978; Needham et al. 2011; Tekin et al. 2011b), and some authors described interindividual variations in the transference of Pb from maternal blood to the placenta (Baghurst et al. 1991; Needham et al. 2011).

A positive correlation between placental and maternal blood Pb has been reported, both in antenatal controls (Baghurst et al. 1991; Lagerkvist et al. 1996) and in specimens collected on the day of birth (Khera et al. 1980; Li et al. 2000; Loiacono et al. 1992; Odland et al. 2001; Roels et al. 1978; Saxena et al. 1994; Schramel et al. 1988). Similarly, placental levels showed a positive correlation with cord blood levels (Baghurst et al. 1991; Loiacono et al. 1992; Needham et al. 2011; Odland et al. 2001; Roels et al. 1978; Saxena et al. 1994; Schramel et al. 1988; Truska et al. 1989; Tsuchiya et al. 1984), with the one exception (Al Saleh et al. 2011). No correlation was observed between placental and maternal milk levels (Schramel et al. 1988).

Effects of collection, processing, or preservation of specimens. Some data indicate that pretreatment of the placenta, elimination of fluids, and freezing may modify Pb levels (Khera et al. 1980). Regarding placental distribution of Pb, Lagerkvist et al. (1996) reported that, in 33% of placentas, Pb concentration differed by a factor of approximately 2 among different lobuli within the same placenta, and Reichrtova et al. (1998a) reported that Pb particulate deposits were more frequently found in syncytiotrophoblasts than at other sites. In contrast, another study failed to detect differences among samples taken from different parts of the same placenta (Piasek et al. 2001). Several studies indicated that Pb levels were higher in amniotic membranes (Baghurst et al. 1991; Fahim et al. 1976; Korpela et al. 1986).

Association between maternal characteristics and exposure to possible risk factors. In a recent study, Tekin et al. (2011b) suggested that differences in placental Pb levels could be influenced by polymorphisms in genes that coded for metallothionein, in a manner similar to that of Cd. With respect to other maternal factors, contradictory results were found regarding the association between placental Pb and maternal age (Khera et al. 1980; Li et al. 2000; Roels et al. 1978; Terrones-Saldivar et al. 2008; Zagrodzki et al. 2003) and with parity (Gundacker et al. 2010; Roels et al. 1978; Singh et al. 2010; Terrones-Saldivar et al. 2008). One study reported an association between Pb and BMI (Zagrodzki et al. 2003).

Of the three metals reviewed, Pb displayed the widest geographical variation: Levels in placentas from normal pregnancies were very high in India (Ahamed et al. 2009; Saxena et al. 1994; Singh et al. 2010) and Saudi Arabia (Al Saleh et al. 2011) and lowest in China (Li et al. 2000; Yang et al. 1997) and Canada (Lafond et al. 2004).

High placental Pb concentrations have been reported in studies of women residing in polluted areas (Baranowska 1995; Zagrodzki et al. 2003) and in the proximity of an e-waste recycling area, with positive correlations between Pb levels and both parents’ length of residence in the area and the father’s e-waste-related work (Guo et al. 2010). Of the four studies that assessed placental Pb in the vicinity of Pb smelters (Fahim et al. 1976; Lagerkvist et al. 1996; Loiacono et al. 1992; Tabacova et al. 1994), only one (which included only pregnant nonsmokers) reported a significant increase in Pb among exposed women (Loiacono et al. 1992). In studies that examined the influence of urban pollution on placental Pb levels, some authors found no association (Roels et al. 1978; Truska et al. 1989; Zagrodzki et al. 2003), whereas others reported higher Pb levels in urban areas (Falcón et al. 2002; Reichrtova et al. 1998b). Only one study addressed and reported seasonal variations (Wibberley et al. 1977).

Among lifestyle-related habits, reports on smoking and placental Pb yielded inconclusive results: Whereas some researchers described a clear association with smoking (Kutlu et al. 2006; Odland et al. 2001; Stasenko et al. 2010), others failed to confirm these results (Al Saleh et al. 2011; Falcón et al. 2002; Piasek et al. 2001; Roels et al. 1978). There were isolated reports of a negative correlation between placental Pb and maternal poultry and mushroom consumption (Gundacker et al. 2010) or with daily coffee consumption (Al Saleh et al. 2011), as well as a positive association with ingestion of tap water containing Pb concentrations > 50 μg/L (Hubermont et al. 1978). In addition, Pb levels might be related to wine consumption before (Osman et al. 2000) but not during pregnancy (Gundacker et al. 2010; Yang et al. 1997). Increased placental Pb levels were also present in long-term pottery industry workers, and painters in particular (Khera et al. 1980).

Possible effects of Pb on the placenta or pregnancy. Several studies found an association between placental Pb levels and some gestational problems. Whereas data on pregnancies with early membrane ruptures were inconclusive, consistently high placental Pb levels—not always statistically significant—were reported for stillbirths (Baghurst et al. 1991; Khera et al. 1980; Wibberley et al. 1977), neonatal deaths (Falcón et al. 2003a; Wibberley et al. 1977), and malformed infants (Wibberley et al. 1977), as well as for women reporting previous miscarriages (Gundacker et al. 2010). Although some researchers observed high Pb levels in placentas from preterm deliveries (Ahamed et al. 2009; Baghurst et al. 1991; Fagher et al. 1993; Fahim et al. 1976), overall, placental Pb was not associated with gestational age (Gundacker et al. 2010; Guo et al. 2010; Loiacono et al. 1992; Roels et al. 1978) except in one study (Falcón et al. 2003a).

The relationship of Pb with birth weight also yielded contradictory results. Most studies observed no association (Falcón et al. 2003a; Guo et al. 2010; Loiacono et al. 1992; Roels et al. 1978; Tekin et al. 2011b), but two found a positive correlation between Pb levels and birth weight (Al Saleh et al. 2011; Gundacker et al. 2010). In contrast, other authors reported an inverse correlation (Llanos and Ronco 2009; Stasenko et al. 2010), with higher levels of Pb in placentas of low-birth-weight babies (< 2500 g) (Llanos and Ronco 2009). In addition, pregnancies with IUGR had placental levels that were high but not statistically significant (Klapec et al. 2008).

Of the studies that examined whether Pb-related gestational problems might be linked to oxidative stress measured via placental biomarkers, a positive association in preterm gestation was reported by Ahamed et al. (2009), but Llanos and Ronco (2009) observed no correlation in neonates with low birth weight for gestational age. Finally, one study showed that placental Pb levels correlated negatively with activity of steroid sulfatase, an enzyme implicated in the metabolism of steroids (Karp and Robertson 1977).

## Discussion

In this review we provide a structured breakdown of the results of studies published from 1976 through 2011 on Hg, Cd, and Pb levels in human placentas. We found that the use of the placenta as a biomarker of exposure to heavy metals is not properly developed because of the heterogeneity among the studies. This heterogeneity applies not only to the populations selected but also to the processing of specimens and presentation of results.

Most of the studies reporting placental levels of these trace metals were conducted on small convenience samples of healthy pregnant women, and the authors did not always describe the inclusion/exclusion criteria they used. These factors limit the external validity of the results. Furthermore, adjusted analyses considering basic potential confounders, such as age or smoking, were not always provided. Specific aspects of these studies, such as type of birth, must be deemed confounding factors. The different ways of obtaining placentas in C-sections and vaginal births, taken together with the circumstances that indicate which technique was used, might result in differences unrelated to environmental exposures.

The lack of universally accepted protocols for collecting and processing placental specimens also hinders comparison of the results. The procedure for collection, pretreatment, storage, and preparation of the placenta prior to analysis varied among studies, although available data suggest that such processes may influence the magnitude and comparability of the trace metal concentrations (Khera et al. 1980; Lagerkvist et al. 1996). Particularly relevant is the representativeness of the placenta specimen vis-à-vis the organ as a whole. In many studies, a single spot tissue specimen was obtained. In practice, this implies an assumption that trace metals are uniformly distributed throughout the placenta. Nevertheless, published data, particularly in the case of Cd and Pb, question this assumption. To solve this problem, some authors have suggested homogenizing the complete organ or instead obtaining and then aggregating and homogenizing random specimens of multiple parts of the placenta (Iyengar and Rapp 2001a). With regard to the methodology used for laboratory analysis, our search was confined to studies undertaken in 1976 and later, a time when fundamental analytical techniques, such as nuclear activation and atomic absorption spectrophotometry in particular, were being developed for the purpose of obtaining reliable results in the quantification of trace metals in placental tissue (Iyengar and Rapp 2001b). Nevertheless, we failed to locate studies that compared the performance of the various analytical approaches specifically in placental tissue. Thus, we cannot rule out possible variability over time attributable to the different analytical methods used and the fine-tuning and improvement of the related quality-control measures.

Because our review was limited to studies published in English or Spanish, results published in other languages are underrepresented. In addition, we were unable to retrieve the full text of some studies (mainly nonindexed), even after requesting copies directly from the authors. A further limitation is that the heterogeneity of the original studies prevented us from providing any reliable quantitative summary measure. Despite the limitations of validity and comparability, some conclusions can be drawn.

Few studies reported total Hg in placentas. Existing studies show a good correlation between Hg levels in placenta and commonly accepted biomarkers of children’s exposure, such as Hg in maternal and newborn blood and hair. Perhaps the existence of these classic indicators justifies in part the limited interest shown in placental Hg. Although higher Hg levels were found in Asian countries, the lack of association with dietary fish intake in the few studies conducted on women with moderate consumption might generate doubts about its possible utility as a biomarker of exposure in these settings. The role of placenta as a barrier for this metal is not yet clear, and even though the Hg level might have some effect on normal functioning of the placenta, it does not seem to be associated with deleterious effects on pregnancy.

Extracted data indicate that Cd accumulates in the placenta; this may explain why placental levels usually correlate with maternal but not cord blood levels. However, if Cd levels in the placenta, as well as the placenta’s efficacy as a barrier, are modulated by maternal polymorphisms linked to metallothionein, this information should be considered in the assessment of this biomarker in terms of exposure. Clarification is also needed with respect to the possible existence of a saturation threshold above which the placenta becomes unable to accumulate Cd, because this may limit the utility of specimens in environmental monitoring. The single determinant most clearly associated with this metal is exposure to smoking during pregnancy. Accordingly, it is essential that studies furnishing information on placental Cd levels give detailed information on the smoking habits of the women and provide separate estimators for nonsmokers. Reported results also suggest a possible relationship between Cd and IUGR or low anthropometric neonatal parameters, which need to be confirmed by additional research.

Pb was the pollutant that presented the clearest geographic differences in placental concentration. The wide range of values seen for this metal in the placenta suggests that the placenta could be a good choice for biomonitoring environmental Pb exposure. Available studies showed that Pb crosses the placenta but does not accumulate. They also suggested an association between placental Pb levels and some gestational disorders, such as stillbirths; however, the data were contradictory and failed to clarify a possible relationship with low birth weight, IUGR, or prematurity.

The associations between placental metal levels and birth weight or gestational age could be biased because exposure to some pollutants might be related to spontaneous abortion, as could also be possible in women with high Pb levels (Wibberley et al. 1977). Prospective studies of maternal exposure early in pregnancy could clarify these issues and evaluate whether pregnancy-related diseases may cause metal accumulation in placental tissue. None of the reviewed studies focused on the association between placental levels and any long-term effect. This is particularly relevant in comparing the utility of placental determinations of Hg or Pb in terms of biomonitoring because associations between children’s neurological development and levels of these metals have indeed been described (Wigle et al. 2008).

In our opinion, international biomonitoring committees should propose compulsory requirements and quality standards for all research involving the use of placentas to measure exposures. Studies should be required to have an appropriate epidemiologic design that is focused on ensuring the representativeness of the target population. They must clearly define inclusion/exclusion criteria and collect data on maternal (sociodemographic, clinical, pollutant-related exposures) and gestational characteristics (complications during pregnancy, gestational age, type of birth, seasonality, result of gestation, and birth weight). Tissue collection, preservation, and processing of specimens likewise require standardization. In biomonitoring studies, initial washing of the placenta to remove blood, followed by homogenization of the whole organ, might enable more comparable results to be obtained by researchers. However, sampling of specific parts of the placenta could be useful for research focused on understanding metal deposit in this organ, as well as the possible association between metal concentration and gestational problems.

Quality assurance procedures should be applied to laboratory processes. Among the available techniques, those with low detection limits (e.g., graphite furnace–atomic absorption spectrometry, inductively coupled plasma–mass spectrometry) might be especially suitable for measuring metal levels in the general population in low-exposure environments. Finally, all of this information should be adequately described in the study reports. We recommend that metal levels be reported in standardized units (e.g., in nanograms per gram wet weight) and that appropriate descriptive statistics and their corresponding confidence intervals be combined with multivariate analyses to identify real risk factors associated with high levels of these pollutants.

## Conclusions

Although interest in the study of heavy-metal levels in the placenta has increased in recent decades, published studies are excessively heterogeneous. Standardized criteria for placenta collection, preservation, processing, and analysis are therefore needed to ensure that comparable results can be obtained. This will allow clarification of the possible effects of these metals in pregnancy, as well as the incorporation of placental tissue into environmental monitoring systems based on human biological samples.

## Supplemental Material

(324 KB) PDF
