# Supplementary material for: Mercury, Cadmium, and Lead Levels in Human Placenta: A Systematic Review
Source: Environ Health Perspect. 2012 May 16;120(10):1369–77. doi: 10.1289/ehp.1204952 (PMC3491942; doi:10.1289/ehp.1204952)
Supplement: (324 KB) PDF [file ehp.1204952.s001.pdf]

Supplemental Material.

Mercury, Cadmium and Lead Levels in Human Placenta: a Systematic Review.

Authors: María D. Esteban-Vasallo, Nuria Aragonés, Marina Pollan, Gonzalo López-Abente, Beatriz Pérez-Gómez.

TABLE OF CONTENTS

Supplemental Material, Figure S1: Flow diagram of study selection process....2

Supplemental Material, Figure S2: Number of studies published with data on trace metals in placenta: overall and with a breakdown by metal studied (1976-2011)....2

Supplemental Material, Table S1. Studies ascertaining total Mercury, Cadmium or Lead levels in placenta (1976-2011). Main characteristics. 3

Supplemental Material, Table S2. Studies on total Mercury(Hg) levels in placenta (1976-2011): main results (in ng/g wet weight) in chronological order....7

Supplemental Material, Table S3 . Studies on Cadmium (Cd) levels in placenta (1976-2011): main results (in ng/g wet weight) in chronological order....9

Supplemental Material, Table S4. Studies on Lead (Pb) levels in placenta (1976-2011): main results (in ng/g wet weight) in chronological order....13

Supplemental Material, Reference list....17

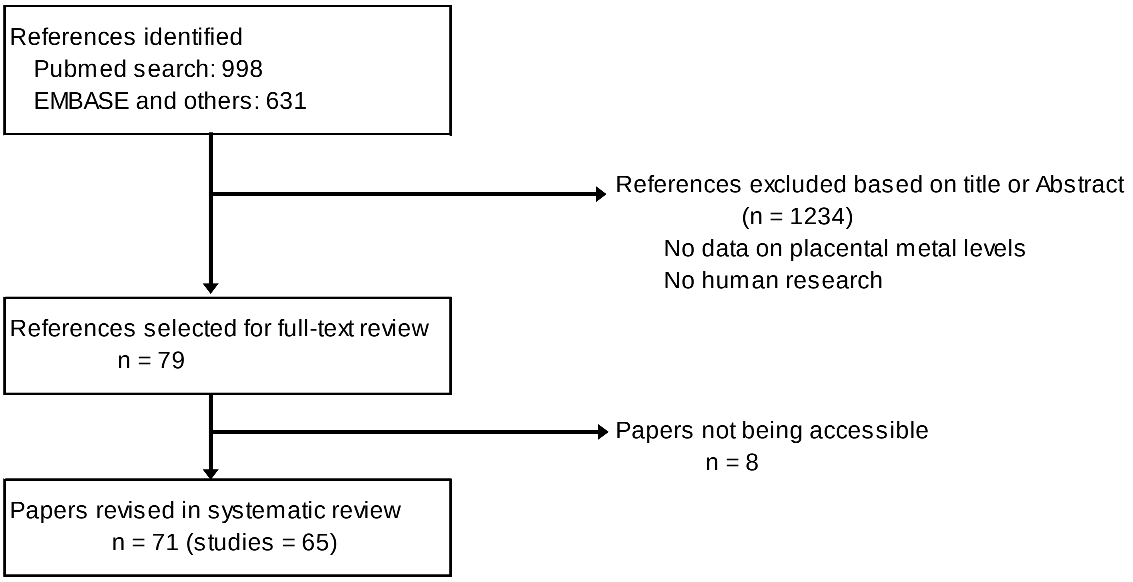

Supplemental Material, Figure S1: Flow diagram of study selection process

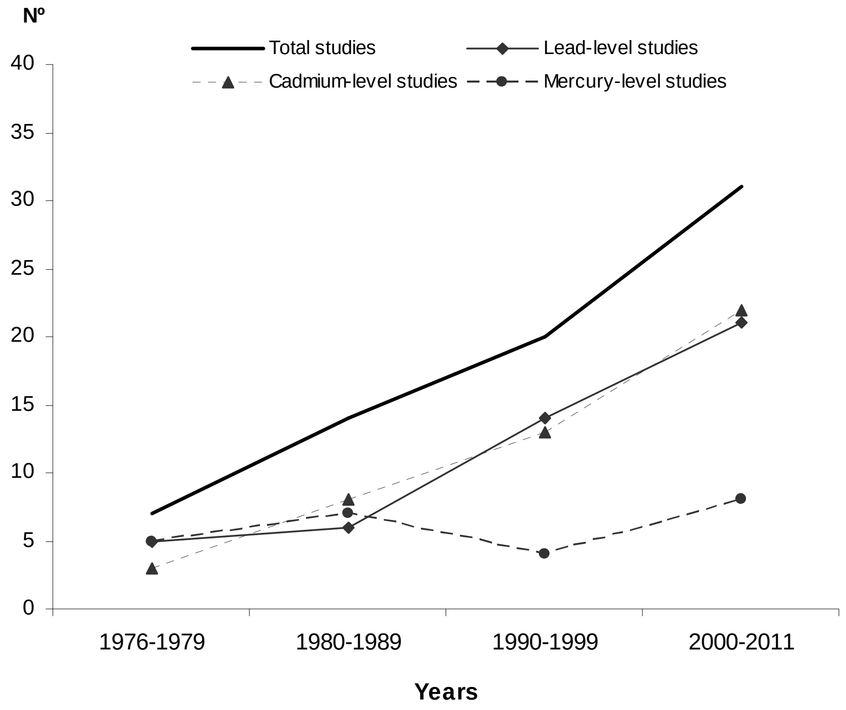

Supplemental Material, Figure S2: Number of studies published with data on trace metals in placenta: overall and with a breakdown by metal studied (1976-2011)

Supplemental Material, Table S1. Studies ascertaining total Mercury, Cadmium or Lead levels in placenta (1976-2011). Main characteristics.

Author, year, country	Metal	Nº	Pop. Sample Method	Participants	IC	Ethic Com.	Other biomarkers	Placenta preparation	Analit. Method	LOD	QQ
Fahim et al.1976<br>USA (Missouri)	Pb	502	CS	Pregnant women from two regions with and without lead mining activity	No	No	Cord blood<br>Cord tissue<br>Maternal blood	Samples were obtained at a distance of one inch from the right side of the cord	CRAAS	No	No
Glaster et al.1976<br>USA (Alaska)	Hg	18	CS	Eskimo mothers selected according to residence	Yes	No	Cord blood<br>Maternal blood<br>Maternal hair<br>Breast milk	Placental tissue was homogenized to obtain a representative sample	FAAS	No	Yes
Pitkin et al.1976<br>USA (Iowa)	Hg	38	ConS	Normal vaginal deliveries at term in women without known abnormal exposure	No	No	Cord blood<br>Maternal blood<br>Breast milk	Fetal membranes and cord were trimmed and blood clots removed. 6-g aliquot of homogenized whole placenta	AAS	No	No
Karp et al.1977<br>USA (Southeast)	Pb, Cd, Hg	58	NS	Not specified. Deliveries at four hospitals	No	No		Stored at –70ºC, thawed, cotyledons cut away, minced and homogenized	NS	No	Yes
Wibberley et al.1977<br>UK (Birmingham)	Pb	126	CS	European and Asian women with normal, premature, and malformed births, stillbirths and neonatal deaths	No	No		Samples were thawed, excessive blood removed, and the tissue dissolved in Soluene	FAAS	No	Yes
Hubermont et al.1978<br>Belgium (Libramont)	Pb, Cd, Hg	70	CS	Pregnant women living in a rural area free of air pollution	No	No	Cord blood<br>Maternal blood	Single spot specimen of total placenta was collected at delivery	FAAS	No	No
Roels et al.1978<br>Belgium (Antwerp, Brussels, Leuven, Tournai,Vilvoorde)	Pb, Cd, Hg	474	CS	Pregnant European women living in different areas	No	No	Maternal blood	20-30g single spot specimen of whole placenta (with membranes), clots removed. 4 1-g aliquots	FAAS	No	Yes
Khera et al.1980<br>UK (Birmingham)	Pb	45	CS	Mothers employed in the pottery industry	No	No		Placentae stored at -20º C for 6-12 months	GFAAS	No	No
van Hattum et al.1981[b]<br>Netherlands (Amsterdam)	Cd	-	Non Available	During 1978 and 1979 placentae were collected from mothers living in the Amsterdam area	-	-		A sampling strategy was developed based on expected placental distribution patterns	FAAS	-	-
Kuhnert et al.1982<br>USA (Cleveland)	Cd	72	CS	Mothers not selected with regard to prenatal or intrapartum medical problems (smokers or not)	Yes	No		Single spot specimen of placental tissue after perfusion to extrude excess blood	AAS	No	No
Peereboom-S et al.1983<br>Netherlands	Cd	60	CS	Smoking and non-smoking mothers	No	No		Tissue blocks with chorionic plate & decidua basalis (0.5 cm), close to umbilical cord	AAS	No	No
Suzuki et al.1984<br>Japan	Hg	12	CS	10 full-term and 2 pre-term deliveries	No	No		Placenta perfused to extrude blood. Chorionic tissue from 1-2 cotyledons	AAS	Yes	No
Tsuchiya et al.1984<br>Japan (Nagoya)	Pb, Cd, Hg	110<br>113<br>210	ConS	Normal deliveries. 1974-78. Industrial urban pollution homogenized	No	No	Cord blood<br>Cord tissue<br>Maternal blood	Placenta washed to remove blood and homogenized, 5-g sample	AFAAS	No	No
Capelli et al.1986<br>Italy (Genoa)	Hg	22	ConS	Healthy mothers. Normal deliveries	No	No		Stored at –25º C, defrosted, homogenized, 4- to 6-g sample	CVAAS	Yes	Yes
Korpela et al.1986<br>Finland	Pb, Cd	6	CS	Deliveries at term. Healthy children	No	No		Placenta stored at -20º C. At least four replicated determinations with each sample	AAS	No	Yes
Mayer-Popken et al.1986<br>Germany	Pb	1	CS	Female worker suffering lead poisoning	No	No		Non specified	ETAAS	No	No
Ward et al.1987 [b]	Hg	100	Non Available		-	-			INAA	-	-
Horvat et al.1988 [b]<br>Yugoslavia	Hg	34	Non Available		-	-			INAA	-	-
Kuhnert et al.1988<br>USA (Cleveland)	Cd	249	ConS	Mothers not selected with regard to prenatal or intrapartum medical problems (smokers or not)	Yes	No		Samples of several cotyledons after removing excess blood, membranes & vessels	AAS	No	No
Schramel et al.1988<br>Germany (Munich)	Pb, Cd, Hg	33/26	NS	NS	No	No	Cord blood<br>Maternal blood<br>Breast milk	Single spot specimen from cotyledons (20-30g), stored at -25º C	DPASV<br>CVAAS	Yes	No
Truska et al.1989<br>Czech Republic	Pb, Cd, Hg	100	CS	Women residing in an urban industrial area and semirural area controls	No	No	Cord blood<br>Maternal blood	No pretreatment. 5-g aliquots	FAAS<br>CVAAS	No	Yes
Baghurst et al.1991<br>Australia (Port Pirie)	Pb	86	ConS	Subcohort of residents in a lead-smelter town and its surrounding rural areas	No	No		Stored at – 20ºC. Membranes & body minced and homogenized separately. 4 aliquots	ETAAS	No	Yes
Berlin et al.1992 [b]<br>Sweden	Cd	27	Non Available	Female workers at a nickel-cadmium battery factory	-	-			AAS	-	-

Supplemental Material, Table S1 (cont). Studies ascertaining total Mercury, Cadmium or Lead levels in placenta (1976-2011). Main characteristics

Author, year, country	Metal	Nº	Pop. Sample Method	Participants	IC	Ethic Com.	Other biomarkers	Placenta preparation	Analit. Method	LOD	QQ
Loiacono et al.1992 Yugoslavia (Titova Mitrovica, Pristina)	Pb, Cd	161	ConS	Non-smoking residents in a lead-smelter town and non- exposed controls. Healthy, single births	No	No	Cord blood Maternal blood	Membranes trimmed. 5 to 10 pieces from the mid-disc region, frozen	AAS	No	No
Moberg et al.1992 Sweden (Karlstad)	Cd	38	CS	Single births. Primiparous mothers aged 20-35 years	Yes	Yes		Placenta stored at –20ºC. 4 samples from centre to periphery	AAS	No	Yes
Radomanski et al.1992 [b] Poland	Pb	127	Non Available		-	-				-	-
Soria et al.1992 Spain (Seville)	Hg	27	Vol	Volunteers aged 20-40 years. None had received mercurial medication or used hair dyes or bleaches	No	No	Cord blood Maternal blood Maternal hair	Single spot specimen of cotyledons (5g), perfused to obtain blood-free tissue	CVAAS	No	No
Fagher et al.1993 Sweden (Lund), Poland (Bialystok)	Pb, Cd	28/24	CS	Deliveries by cesarean section. Residents in town and surrounding rural area	Yes	No		3-g single spot specimen	AAS	Yes	Yes
Fréry et al.1993 France (Paris)	Cd	102	CS	Vaginal deliveries. Single healthy children	Yes	No		Two samples (10 g) stored at -70º, washed	GFAAS	No	No
Saxena et al.1994 India (Lucknow)	Pb	217	ConS	Normal and abnormal deliveries. Women non-smokers, unexposed to lead	Yes	No	Cord blood Maternal blood	Fragments of placenta, blood clots removed	DCPES	No	Yes
Tabacova et al. 1994 Bulgaria	Pb, Cd	46	ConS	Residents near a copper smelter and residents in a control area	No	No	Cord blood Maternal blood	Placental samples (single lobules) sectioned from maternal surfate through to the chorionic plate; decidua basalis and chorionic plate cut hawai afterwards. Cleansed of blood and frozen at -40º 1 week	ETAAS	No	Yes
Baranovska 1995 Poland (Silesia)	Pb, Cd	24	ConS	Healthy full-term newborns in residents of a heavily polluted area	No	No	Cord blood Maternal blood	Single spot specimen from cotyledon (20-30 g), stored at - 20º C	GFAAS	No	No
Díaz-Barriga et al.1995 [b] Mexico	Pb, Cd	37	Non Available	Mothers from agricultural zone and smelting and metallurgy activities area	-	-			GFAAS	-	-
Baranowski et al.1996 Poland (Silesia)	Pb, Cd	28	NS	Healthy full-term newborns in residents of a heavily polluted area (Upper Silesia)	No	No		Samples (approx. 20g each) stored at - 20º C	DPV	No	No
Centeno et al.1996 USA	Pb, Cd	25	ConS	Patients soon after delivery, 6 Menke's patients	No	No		Tissue was processed by sampling umbilical cord, membrane & parenchyma, stored al -72º	FAAS	No	No
Lagerkvist et al.1996 Sweden	Pb, Cd	102	CS	Residents near a copper and lead smelter and unexposed controls	No	No		Stored at – 20º C. 6 samples from different lobuli, minced, mixed. 2 samples (1-5 g)	GFAAS	Yes	Yes
Yang et al.1997 China (Shangai)	Pb, Cd, Hg	17/17 18	CS	Non-smoking parturient women occupationally exposed to Hg vapor, and unexposed controls	No	No		Stored at –20º C. 0.1 to 1 g spot specimen near umbilical cord, minced and washed	CVAAS	No	No
Fiala et al.1998 Czech Republic (Brno, Znojmo)	Cd	688	ConS	Deliveries from January through June 1992	No	No		3-5 samples from various parts of each placenta stored at –20ºC, lyophilized (0.2 g)	GFAAS	No	No
Klopov 1998 Russia (Arctic)	Pb, Hg	42	ConS	Nonindigenous women residing in the Russian Artic, the most industrialized artic zone	No	No	Cord blood Maternal blood Breast milk	Not specified	FAAS CVAAS	No	No
Reichrtova et al.1998a,b Slovakia (Bratislava, Spisska Nova Ves, Krompachy, Stara Lubovna)	Pb, Cd	200	RS	Full-term deliveries from industrial and rural regions. Not occupationally exposed	No	No		1-cc samples from marginal, intermediate and periumbilical zones	FAAS	No	No
Scaal et al.1998 Germany (Southwest)	Hg	59	CS	Fetuses with malformations of unknown origin (chromosomal aberrations excluded)	No	No		60 mg of a single spot specimen. Paraffin-embedded tissue, analyzed in duplicate	CVAAS	Yes	Yes
Richter et al.1999 [b] Czech Republic	Pb		Non Available		-	-				-	-
Bush et al.2000 UK	Cd	53	CS	Caucasian women, uncomplicated pregnancies, full-term vaginal or cesarean-section deliveries	No	No	Cord blood Maternal blood	Systematic random samples of placental tissue washed, minced	ICP-MS	Yes	Yes
Kantola et al.2000 Finland (Kuopio) Estonia (Tallinn, Rakvere), Russia (St. Petersburg	Cd	180	Vol	152 healthy volunteers. Normal deliveries with healthy babies and 64 abortions (social reasons)	Yes	No		Frozen –20ºC, cut in pieces, removed outer integuments, 20-g samples homogenized	GFAAS	Yes	Yes

Supplemental Material, Table S1 (cont). Studies ascertaining total Mercury, Cadmium or Lead levels in placenta (1976-2011). Main characteristics

Author, year, country	Metal	Nº	Pop. Sample Method	Participants	IC	Ethic Com.	Other biomarkers	Placenta preparation	Analit. Method	LOD	QQ
Li et al.2000<br>China (Shangai)	Pb	153	CS	Parturient women not occupationally exposed	No	No		NS	AAS	No	Yes
Osman et al.2000<br>Sweden (Solna)	Pb, Cd	89<br>106	P	Pregnant women recruited from October 1994 through January 1996	No	No	Cord blood<br>Maternal blood	Trophoblastic tissue homogenized (decidua basalis & chorionic plate away). 2 samples	ICP-MS	Yes	Yes
Zadorozhnaja et al.2000<br>Ukraine (Kyiv, Dniprodzerzhinsk)	Pb, Cd, Hg	200	CS	Women from two industrialized cities, chosen at random, except for age. Simple births	No	No		Frozen. 3 samples (5g) from center, para- & margin (cotyledons avoided), homogenized	GFAAS<br>CVAAS	Yes	Yes
Odland et al.2001/2004<br>Russia (Nikel, Monchegorsk, Arkhangelsk),<br>Norway (Kirkenes, Hammerfest, Bergen)	Pb, Cd	263	ConS	Women from Arctic and sub-Arctic regions	Yes	No	Cord blood<br>Newborn urine<br>Maternal blood<br>Maternal urine	Decidua basalis & chorionic plate trimmed off 3 cubes from peri-insertional tissue, stored at -20ºC, homogenized and –70ºC	ETAAS	Yes	Yes
Pereg et al.2001<br>Canada (Quebec)	Pb, Cd	40	CS	Caucasians from a coastal town	Yes	No		Connective tissue & blood vessels removed. 10-g sample, homogenized, stored at –80ºC	ICP-MS	Yes	Yes
Piasek et al.2001<br>Croatia (Zagreb)	Pb, Cd	51	CS	Urban, healthy parturients. Normal pregnancies and deliveries at term	Yes	No		Stored –20ºC. 3 samples (excluding chorionic plate & decidua basalis), centre & periphery	ETAAS	Yes	Yes
Falcón et al.2002/2003a,b<br>Spain (Murcia)	Pb, Cd	86/89	CS	Healthy women. Single normal births, non-occupationally exposed	No	No		Stored at –50ºC, trophoblastic tissue with no signs of calcification. 2 3-g samples	GFAAS	Yes	Yes
Osada et al.2002<br>Japan (Chiba)	Cd	51	CS	Healthy non-smoking women. Intrauterine growth restriction and controls	Yes	No		Decidua basalis and chorionic plate trimmed off 2 cubes stored at –45ºC. 20 mg sample	ICP-MS	Yes	Yes
Zagrodzki et al.2003<br>Poland (Krakow, Bieszczady)	Pb	23	CS	Healthy non-smokers in a heavily industrialized area and rural controls. Normal deliveries	No	No		Frozen, washed. 2 samples (0.3-0.6g) from a central portion	GFAAS	No	Yes
Lafond et al.2004<br>Canada (Quebec)	Pb	30	P	Healthy women with no occupational exposure single uncomplicated pregnancies	No	No	Cord blood<br>Maternal blood	Amnion, chorion and decidua were removed, villous tissue cut into 1-inch pieces, washed	ICP-MS	Yes	Yes
Zhang et al.2004<br>China (Hubei)	Cd	44	CS	Soil highly contaminated with Cd. Healthy women aged 20 to 34 years, living at least 15 years in the area. Not occupationally exposed; single births	No	No	Cord blood<br>Maternal blood	2 samples from different sections, stored at -20ºC	ICP-MS	Yes	Yes
Ronco et al.2005a,b<br>Chile (Santiago)	Cd	40	CS	Healthy young parturients with normal pregnancies	No	Yes		Stored at –70ºC, half of semi-thawed placenta washed, lyophilized. 3 decidua & 3 chorionic plate samples homogenized	GFAAS	No	Yes
Hsu et al.2006<br>Taiwan (Taipei)	Hg	46	CS	Pregnant women residing in the city. Deliveries from July 2004 through March 2005	Yes	No	Cord blood<br>Maternal blood	0.5-g spot specimen analyzed in triplicate	Hg analyzer	No	Yes
Kutlu et al.2006<br>Turkey	Pb, Cd	190	ConS	Pregnant women exposed and unexposed to smoking	No	No		Stored at –70ºC. 2 samples (3-5g) homogenized	HHDE	Yes	Yes
Marques et al.2007<br>Brazil (Porto Velho)	Hg	100	CS	Healthy women, willing to breast feed	Yes	Yes	Cord blood<br>Newborn hair<br>Maternal blood<br>Maternal hair	3 aliquots from a single spot from each placenta, stored at -20ºC	CVAAS	No	Yes
Sorkun et al.2007<br>Turkey (Denizli)	Cd	92	CS	Normal term pregnancies; 2nd and 3rd trimesters in winter. Vaginal or cesarean-section deliveries	No	No		Single spot sample from each placenta, stored at -80ºC	GFAAS	No	Yes
Klapec et al.2008<br>Croatia (Osijek)	Pb, Cd	85	CS	Healthy, non-smoking mothers; normal pregnancy & intrauterine growth restriction cases	Yes	No		Stored at –20ºC, connective tissue removed, several small portions combined. 3-g sample	GFAAS	Yes	Yes
Terrones et al.2008<br>Mexico (Aguascalientes)	Pb, Cd	40	CS	Normal pregnancies, & with severe oligoamnios. Vaginal or cesarean-section deliveries	Yes	No		Duplicated sample	GFAAS	Yes	No
Ahamed et al.2009<br>India (Lucknow)	Pb	60	CS	Pre- and full-term vaginal deliveries. Healthy babies; industrial pollution area	Yes	Yes		25g of trophoblastic placental tissue taken from a single spot, frozen at -80ºC, washed. 1-g sample	FAAS	Yes	Yes
Llanos et al.2009<br>Chile (Santiago)	Pb, Cd, Hg	40	CS	Young, healthy, non-smoking mothers with full-term pregnancies; babies with fetal growth restriction or normal birth weight	Yes	Yes		Stored at –70ºC, half of semi-thawed placenta washed, lyophilized & homogenized	AFAAS<br>GFAAS<br>INAA	No	Yes

Supplemental Material, Table S1 (cont). Studies ascertaining total Mercury, Cadmium or Lead levels in placenta (1976-2011). Main characteristics

Author, year, country	Metal	Nº	Pop. Sample Method	Participants	IC	Ethic Com.	Other biomarkers	Placenta preparation	Analit. Method	LOD	QQ
Stasenko et al.2010 Croatia (Zagreb)	Pb, Cd	208	ConS	Healthy mothers, uncomplicated pregnancies, who delivered vaginally at term. 1ª-2ª gestation	Yes	Yes		Stored at –20ºC. 2 samples (central and marginal) excluding chorionic plate	ETAAS	No	Yes
Al-Saleh et al.2010 Saudi Arabia (Al-Kharj)	Pb, Cd, Hg	1576 1578 1568	P	Hospitalized for delivery; residents for a minimum of one year without diabetes or heart problems	Yes	Yes		3 samples (5g each) from various sites, stored at –20ºC, pooled and homogenized	GFAAS CVAAS	Yes	Yes
Grant et al.2010 [b] Jamaica	Hg	52	Non Available	Jamaican mothers with a mean age of 29 years delivering singleton neonates	-	-		Samples were collected and stored at –20ºC, dried and analyzed	INAA	-	-
Gundacker et al.2010 Austria (Vienna)	Pb, Hg	31	CS	Healthy mothers (women with gestational complications dropped out).	Yes	Yes	Cord blood Meconium Maternal blood Maternal hair Breast milk	Stored at –20ºC. 6x6-mm piece cut out of the middle of the placenta & homogenized	GFAAS CVAAS	Yes	Yes
Guo et al.2010 China (Guiyu, Chaonan)	Pb, Cd	220	CS	Mothers from an e-waste recycling area and controls for comparison	Yes	Yes		2x2-cm piece, central region, stored at –20ºC. Cubes of 0.5-g fresh tissue wt	GFAAS	No	Yes
Kippler et al.2010 Bangladesh (Matlab)	Cd	44	CS	Women who had participated in a nutrition intervention trial in a previous pregnancy	Yes	Yes	Cord blood Maternal urine	Drained of blood. Stored at -20ºC. 2 pieces of trophoblastic tissue frozen at –20ºC	ICP-MS	Yes	Yes
Singh et al.2010 India (Lucknow)	Pb	60	CS	Healthy pregnant women who gave birth to single healthy babies. Not occupationally exposed	Yes	Yes	-	25 g of trophoblastic tissue with no signs of calcification	FAAS	Yes	Yes
Needham et al.2011 Denmark (Faroe I.)	Pb, Cd, Hg	15	ConS	Normal births; 5 mothers with no whale meat or blubber intake. Among the participants of a cohort, 5 accounted for the highest hair-hg, and 5 for the highest milk-PCB concentrations	Yes	Yes	Cord blood Cord tissue Maternal blood Maternal hair Breast milk	Multiple cube-like pieces from peripheral and central lobes, avoiding calcium deposits and selecting trophoblastic tissue from fetal part of placenta	GFAAS CVAAS	Yes	Yes
Tekin et al.2011a,b Turkey (Ankara)	Cd, Pb	91	ConS	Healthy non-smoking mothers living in Ankara for more than 3 years, not occupationally exposed. Vaginal or cesarean-section deliveries	Yes	Yes	Cord blood Maternal blood	Samples stored at –20ºC, two samples from center, four from periphery, excluding chrorionic plate & decidua basalis	GFAAS	No	Yes

[b] Full text was unavailable (data incorporated into the table are obtained from the abstracts or quoted in other papers)

Abbreviations and criteria used in the table

Nº: Number of placentas analyzed for each metal;

Metals: Hg: mercury; Cd: cadmium; Pb: lead

Population Sample method: P: population-based study; CS: convenience sample; ConS: consecutive sampling; RS: randomly selected; Vol: volunteers

IC: Informed consent; Yes indicates that it is specifically mentioned in the report;

Ethic Com: Ethical committee approval; Yes indicates that it is specifically mentioned in the report

Analytical methods: AAS: atomic absorption spectrometry; AFAAS: acetylene-air flame AAS; CRAAS: carbon rod AAS; CVAAS: cold vapor AAS; DCPES: DCP emission spectrophotometry ; DPASV: anodic stripping voltammetry; DPV: pulse differential polarography; ETAAS: electro-thermal atomic AAS; FAAS: flameless AAS; GFAAS: graphite furnace AAS; HGAAS: hydride generation AAS; HHDE: Hanging hg drop electrode; ICP-MS: inductively coupled plasma - mass spectrometry; INAA: instrumental neutron activation analysis; NS: non specified

LOD: limit of detection; Yes indicates that the paper reports the LOD value

QQ: quality control procedures; Yes indicates that any quality control procedures are specifically mentioned in the report.

Supplemental Material, Table S2. Studies on total Mercury(Hg) levels in placenta (1976-2011): main results (in ng/g wet weight) in chronological order

Reference, year country	Group characteristics	n	Arithmetic mean ± SD	Median	Rank	Main results
Glaster et al.1976<br>USA (Alaska)	Rural coast (high fish intake)<br>Rural interior (medium fish intake)<br>Urban (low fish intake)	8<br>3<br>7	38.9 ± 5.1<br>30.7 ± 4.8<br>11.6 ± 1.3			Higher levels than maternal and cord blood. Levels 4-5 fold higher in mothers with daily fish or seal intake. Positive correlation with cord blood levels. Eskimos.
Pitkin et al.1976<br>USA (Iowa)	Population with no abnormal exposure. Rural environment, low fish intake	38	1.4 ± 0.2			Slightly more than one third had no detectable mercury. Higher levels than maternal and cord blood. No correlation with maternal age, parity or birth weight
Karp et al.1977<br>USA (Southeast)	Total<br>Augusta<br>Birmingham<br>Charlotte	58<br>19<br>22<br>17	14.0 ± 1.0<br>8.0 ± 1.0<br>15.0 ± 3.0<br>19.0 ± 3.0			Difference between cities is statistically significant. Mercury level correlates negatively with enzyme activity of isocitric dehydrogenase and steroid sulfatase. This would suggest an inhibitory effect of the metal on enzyme activity
Hubermont et al.1978<br>Belgium (Libramont)	Deliveries in a rural area	70	9.7 ± 9.7	9.2	3.7 - 22.9	Lower levels than maternal and cord blood
Roels et al.1978<br>Belgium (Antwerp, Brussels, Leuven, Tournai,Vilvoorde)	European (excluding Afro-Asians)<br>Smokers (> 3 cigarettes/day)<br>Non-smokers<br>Rural and semirural environment<br>Urban and industrial environment	474<br>109<br>333<br>231<br>236	15.3 ± 14.1<br>12.2 ± 9.8<br>16.2 ± 15.0<br>16.1 ± 16.1<br>14.3 ± 14.3	10.6<br>10.6<br>10.7<br>10.2<br>10.7	1.1 - 103.2<br>1.1 - 89.1<br>1.3 - 103.2<br>1.3 - 103.2<br>1.1 - 89.1	Mercury does not show accumulation in placenta. No relation with maternal and cord blood levels. No significant differences by area of residence or tobacco use. No relation with maternal age, paternal occupation, drinking habits, gestational age, parity and birth weight
Suzuki et al.1984<br>Japan	2 pre- and 10 full-term deliveries	12	53.8 ± 21.3<br>50.7[b]	48.4	34.6 - 104.9	Inorganic significantly higher than organic mercury in placenta. Methylmercury moves freely across the placenta, but inorganic mercury is prevented from been transferred to fetus
Tsuchiya et al.1984<br>Japan (Nagoya)	Urban and industrial environment	210	185.0 ± 452.0		2.0 - 3166	Significantly higher than maternal and cord blood. Positive correlation with cord blood (r:0.595)
Capelli et al.1986<br>Italy (Genoa)	Healthy women; normal deliveries	22	12.7 ± 9.0			65% of total mercury is organic. No correlation with Selenium. Dry/wet ratio: 6.3
Ward et al.1987[a]		100			2.0 - 13.0	
Horvat et al.1988 [a]<br>Yugoslavia		34	12.9 ± 7.7			
Schramel et al.1988<br>Germany (Munich)	Urban environment	26	4.1 ± 2.1			Dry/wet ratio: 6.2
Truska et al.1989<br>Czech Republic	Non-smokers. Industrial area<br>Non-smokers. Semirural area	50<br>50	2.0 ± 0.9<br>2.2 ± 1.0	2.0<br>2.0		Mercury does not accumulate in placenta. Lower levels than maternal and cord blood in erythrocytes and plasma. Positive correlation with maternal erythrocytes in the industrial region
Soria et al.1992<br>Spain (Seville)	Volunteers, aged 20-40 years	27	5.4 ± 3.1		2.3 - 14.3	Positive linear relationship with arterial cord blood. 93.5% is methylmercury
Yang et al.1997<br>China (Shangai)	Occupational exposure (lamp factory)<br>Unexposed controls	9<br>9	127.3 ± 23.9<br>68.1 ± 18.9			Statistically significant differences. Higher levels than maternal and cord blood. Metallic mercury easily transferred through the placenta. No apparent adverse effects were found in babies from exposed group
Koplov et al.1998<br>Russia (Arctic)	Norilsk<br>Salekhard	?<br>?	5.6 ± 0.7<br>7.2 ± 1.2			

Supplemental Material, Table S2 (cont). Studies on total Mercury levels in placenta (1976-2011): main results (in ng/g wet weight) in chronological order

Reference, year country	Group characteristics	n	Arithmetic mean ± SD	Median	Rank	Main results
Scaal et al.1998<br>Germany (Southwest)	Fetuses with malformations of unknown origin (chromosomal aberrations excluded)	59		13.0		Study showed no evidence of Hg implication in these malformations
Zadorozhnaja et al.2000Ukraine (Kyiv, Dniprodzerzhinsk)	Urban area. Industrial pollution	200		<2.2 (LOD)	2.2 - 45.8	28% with detectable mercury levels
Hsu et al.2006<br>Taiwan (Taipei)	Single deliveries July/2004-March/2005	46	19.2 ± 1.8	18.0	6.2 –81.0	Higher levels than maternal and cord blood. Positive correlation with fish consumption prior to but not during pregnancy. Not associated with dental fillings
Marques et al.2007<br>Brazil (Porto Velho)	Healthy mothers, willing to breast feed	100		8.1	0.4 - 56.3	No significant differences by fish consumption. Positive correlation with Hg in maternal (r:0.321) and neonatal (r:0.219) hair, maternal (r:0.250) and cord (r:0.857) blood
Llanos et al.2009<br>Chile (Santiago)	Term neonates with low birth weight<br>Term neonates with normal weight	20<br>20	33.3 ± 5.0<br>33.3 ± 8.3			No statistically significant differences
Al-Saleh et al.2010<br>Saudi Arabia (Al-Kharj)	Women hospitalized for delivery; residents for a minimum of one year	1568	10.7 ± 67.2	5.2	0.0 - 2167.2	Levels of Hg in cord and maternal blood were correlated with those in placenta (r:0.206 and r:0.425, respectively). Maternal Hg, cord Hg and Cd levels and BMI were positively associated with placental Hg levels (B-coefficients: 0.401, 0.129, 0.081 and 0.06). Maternal and placental Cd levels negatively influenced placental Hg levels (–0.067 and –0.085, respectively)
Grant et al.2010 [a]<br>Jamaica	Jamaican mothers, mean age 29 years, delivering singleton neonates	52	7.3			
Gundacker et al.2010<br>Austria (Vienna)	Healthy mothers in urban environment	31		1.9	0.1 - 11.7	Hg retained in substantial amounts in the placenta. Levels were not associated with newborn anthropometry
Needham et al.2011<br>Denmark (Faroe I.)	Normal births in a fishing community	15		87.0		Hg showed excellent correlations between concentrations in placenta and cord tissue. High correlation with cord blood (r:0.97). Ratio to cord blood: 7.2

[a] Full text was unavailable (data incorporated into the table are obtained from the abstracts or quoted in other papers)
[b] Geometric mean

Supplemental Material, Table S3 . Studies on Cadmium (Cd) levels in placenta (1976-2011): main results (in ng/g wet weight) in chronological order

Reference, year country	Group characteristics	n	Arithmetic mean ± SD	Median	Rank	Main results
Karp et al.1977<br>USA (Southeast)	Total<br>Augusta<br>Birmingham<br>Charlotte	58<br>19<br>22<br>17	37.0 ± 3.0<br>53.0 ± 4.0<br>30.0 ± 5.0<br>28.0 ± 4.0			Difference between cities is statistically significant. Cd level correlates positively with enzyme activity of isocitric dehydrogenase and carnitine palmityl-transferase. This would suggest that enzyme activity is enhanced by the metal
Hubermont et al.1978<br>Belgium (Libramont)	Deliveries in a rural area	70	11.4	9.3	3.0 - 37.5	Higher levels than maternal and cord blood
Roels et al.1978<br>Belgium	European (Afro-Asians excluded)<br>All non-smokers<br>rural and semirural area<br>urban and industrial area<br>All smokers (> 3 cigarettes/day)<br>rural and semirural area<br>urban and industrial area	474<br>333<br>166<br>163<br>109<br>47<br>62	13.2 ± 8.7<br>12.5 ± 8.6<br>12.3<br>12.6<br>15.7 ± 9.2<br>13.7<br>17.1	10.8<br>10.5<br>10.5<br>10.3<br>12.7<br>10.4<br>13.7	2.5 - 78.9<br>2.5 - 78.9<br>2.5 - 59.8<br>2.6 - 78.9<br>3.4 - 43.8<br>3.4 - 42.7<br>4.6 - 43.8	Placenta seems to be an efficient barrier for Cd. Concentration 10-fold higher than maternal blood levels. Positive correlation with maternal blood levels (r:0.38). Higher concentrations of Cd in smokers. No significant differences by area of residence. No relation with maternal age, paternal occupation, drinking habits, gestational age, parity and birth weight
van Hattum et al.1981 [a]<br>Netherlands (Amsterdam)	Non-smokers<br>Smokers		8.5 ± 3.3<br>11.0 ± 5.5			
Kuhnert et al.1982<br>USA (Cleveland)	Non-smokers<br>Smokers	31<br>41	13.7 ± 6.4<br>18.1 ± 7.3			Percentage increase in Cd due to smoking, 32% (p<0.01). Placenta acts as a barrier. Higher levels than maternal and cord blood. No correlation with these
Peereboom-S et al.1983<br>Netherlands	Non-smokers<br>All smokers<br>15-25 cigarettes/day<br>20-60 cigarettes/day	30<br>30<br>10<br>20	8.5 ± 3.3<br>11.0 ± 5.5<br>13.7 ± 6.3<br>9.5 ± 4.3			Higher concentrations in smokers. Human placenta could have a limited capacity for Cd. At a higher exposure level -of 25 cigarettes and over- the placenta may become saturated and Cd might leak through the placenta, reaching the fetus
Tsuchiya et al.1984<br>Japan (Nagoya)	Urban and industrial environment	113	30.0 ± 8.0		1.0 - 81.0	Significantly higher than maternal and cord blood. Positive correlation with cord blood (r:0.322. p<0.01)
Korpela et al.1986<br>Finland	Full-term deliveries; healthy neonates	6	20.4 ± 14.4			As a partial barrier for the fetus, the placenta accumulates Cd
Kuhnert et al.1988<br>USA (Cleveland)	Non-smokers nulliparous (first infant)<br>Non-smokers primiparous (second infant)<br>Non-smokers multiparous<br>Smokers nulliparous (first infant)<br>Smokers primiparous (second infant)<br>Smokers multiparous	17<br>28<br>27<br>17<br>33<br>39	≈ 9.3<br>≈ 8.4<br>≈ 9.3<br>≈ 10.5<br>≈ 12.7<br>≈ 14.6			Increased parity related to increased levels of placental Cd in smokers, and decreased placental zinc in smokers and nonsmokers
Schramel et al.1988<br>Germany (Munich)	Urban environment	33	5.0 ± 2.2			Weak correlation with maternal blood (r:0.48). No correlation with cord blood or maternal milk. Dry/wet ratio: 6.2
Truska et al.1989<br>Czech Republic	Non-smokers, industrial area<br>Non-smokers, semirural area	50<br>50	3.2 ± 2.0<br>4.0 ± 3.7	2.0<br>3.5		Cd does not accumulate in the placenta. Lower levels than maternal and cord erythrocytes and plasma. Positive correlation with maternal erythrocytes and plasma
Berlin et al.1992 [a]<br>Sweden	Battery factory workers	27	21.0 ± 22.0		2.0 - 95.0	Morphological and ultrastructural studies of placental tissue showed no effect of Cd. Placental Cd levels were possitively correlated with maternal blood Cd concentrations

Supplemental Material, Table S3 (cont) . Studies on Cadmium (Cd) levels in placenta (1976-2011): main results (in ng/g wet weight) in chronological order

Reference, year country	Group characteristics	n	Arithmetic mean ± SD	Median	Rank	Main results
Loiacono et al.1992<br>Yugoslavia (Titova Mitrovica, Pristina)	Titova Microvica (lead smelter)<br>Pristina (unexposed to lead smelter)	106<br>55	13.7 ± 9.7<br>9.4 ± 3.6			Statistically significant differences. No associations between placental Cd concentrations and birth weight, length of gestation, or placental lead levels
Moberg et al.1992<br>Sweden (Karlstad)	Non-smokers (thiocyanate < 50)<br>Smokers (thiocyanate 50-69)<br>Heavy smokers (thiocyanate ≥ 70)	23<br>12<br>3	3.4 ± 0.3<br>3.8 ± 0.3<br>6.0 ± 1.1			Higher levels in smokers. Placental Cd concentration was nearly half in women who had consumed less grain fiber or had higher iron status in late pregnancy
Fagher et al.1993<br>Sweden (Lund),<br>Poland (Bialystok)	Pre-term cesarean section<br>Full-term cesarean section	13<br>11	33.3 ± 33.3<br>16.7 ± 33.3			No statistically significant differences. Significantly higher levels in Polish women. No differences between smokers and non-smokers
Fréry et al.1993<br>France (Paris)	Parenchyma with no calcifications<br>Parenchyma with calcifications	74<br>28		8.5<br>11.8		Significantly higher in cases of parenchymal calcifications (p<0.05), even after taking smoking habits and gestational age into account
Baranovska 1995<br>Poland (Silesia)	Heavily polluted area	24	18.3 ± 11.7	16.7	1.7 - 50.0	Placenta acts as a barrier. High levels in maternal though not in cord blood
Díaz-Barriga et al.1995 [a]<br>Mexico	Agricultural zone<br>Smelting and metallurgy activities area	16<br>21	1.3 ± 0.5<br>10.9 ± 2.0		LOD - 7.1<br>LOD - 33.5	
Baranowski et al.1996<br>Poland (Silesia)	Healthy full-term newborns in residents of a heavily polluted area (Upper Silesia)	28	13.0			Placenta is a better barrier for Cd than for Pb
Centeno et al.1996<br>USA	Patients soon after delivery,<br>6 Menke's patients	25	4.4 ± 3.0<br>6.3 ± 1.2		0.4 - 9.1<br>3.0 - 4.7	
Lagerkvist et al.1996<br>Sweden	Area exposed to lead smelter<br>Unexposed area	49<br>53	3.6 ± 2.8<br>4.9 ± 4.2			Higher levels in smokers. Cd accumulates in the placenta. Levels 4-6 fold higher than in maternal and cord blood. Uniform distribution. Correlation with maternal blood (r:0.39)
Yang et al.1997<br>China (Shangai)	Occupational exposure (lamp factory)<br>Unexposed controls	9<br>9	1.3 ± 0.9<br>1.2 ± 0.8			No statistically significant differences
Fiala et al.1998<br>Czech Republic<br>(Brno, Znojmo)	Urban areas	688	3.0 ± 7.4	2.1	0.0 - 153.0	Higher levels with higher maternal age (≥27 vs. ≤ 21 years). Age and smoking habit related with decrease in Zn/Cd rate
Reichrtova et al.1998a,b<br>Slovakia (Bratislava, Spisska Nova Ves, Krompachy, Stara Lubovna)	Industrially polluted region, heavy traffic<br>Iron mining region, intermediate traffic<br>Small city, copper mining, light traffic<br>Rural region, no industry	50<br>50<br>50<br>50	17.3 ± 22.8<br>22.1 ± 11.2<br>15.0 ± 14.1<br>15.4 ± 13.2	10.5<br>18.6<br>10.7<br>11.4	23.0 - 1610.0<br>85.0 - 493.0<br>20.0 - 90.0<br>5.0 - 867.0	Statistically significant differences. Placental cadmium concentrations gave a better reflection of smoking habit than environmental pollution
Bush et al.2000<br>UK	Non-smokers (< 5 cigarettes/day)<br>Smokers (≥ 5 cigarettes/day)	33<br>20	11.3 ± 0.8<br>15.5 ± 1.6			Statistically significant differences. Positive correlation with number of cigarettes. Morphological changes in placenta of smoking women, suggesting hypoxia
Kantola et al.2000 [b]<br>Finland (Kuopio), Estonia (Tallinn, Rakvere)<br>Russia (St, Petersburg)	Non-smokers (> 1 year), full-term deliveries<br>Smokers, normal full-term deliveries<br>Non-smokers (> 1 y), 1st trimester miscarriage<br>Smokers, 1st trimester miscarriage	106<br>26<br>23<br>25	3.7 ± 1.9<br>4.6 ± 2.0<br>0.8 ± 1.2<br>1.7 ± 2.9			Statistically significant differences between smokers and non-smokers in the first trimester (p=0.006) and at term (p=0.015), and between first trimester and term levels. Not associated with maternal age or birth weight. Differences by region in non-smokers (> in St. Petersburg)
Osman et al.2000<br>Sweden (Solna)	Deliveries in Solna	106	5.6	5.2	1.1 - 19.1	Higher levels of Cd in placenta and maternal (though not cord) blood of smokers

Supplemental Material, Table S3 (cont) . Studies on Cadmium (Cd) levels in placenta (1976-2011): main results (in ng/g wet weight) in chronological order

Reference, year country	Group characteristics	n	Arithmetic mean ± SD	Median	Rank	Main results
Zadorozhnaja et al.2000<br>Ukraine (Kyiv, Dniprodzerzhinsk)	Urban area. Industrial pollution	200		5.2	<1.8 - 20.8	No differences with smoking habit (N=8), maternal age or parity
Odland et al.2001/2004<br>Russia (Nikel, Monchegorsk, Arkhangelsk),<br>Norway (Kirkenes, Hammerfest, Bergen)	All women (Arctic and sub-Arctic regions)<br>Russian women<br>Norwegian women	263<br>113<br>150	5.8 ± 3.2<br>6.2 ± 2.8<br>5.5 ± 3.2	5.3<br>5.8<br>4.8	1.8 - 33.5<br>2.2 - 19.7<br>1.8 - 35.0	Statistically significant differences between Russian and Norwegian women. No correlation with tobacco. Associated with maternal blood levels (p<0.005)
Pereg et al.2001<br>Canada (Quebec)	Non-smokers<br>Smokers (3 - 25 cigarettes/day)	23<br>17	6.0<br>12.0			Statistically significant differences. Correlation with daily smoking (r:0.83). No relation with DNA adducts
Piasek et al.2001<br>Croatia (Zagreb)	Non-smokers (> 1 year)<br>Smokers	24<br>27	16.4 ± 1.7<br>29.7 ± 2.5			Statistically significant differences. No differences between central and peripheral samples of placenta. Lower iron in placenta of smokers
Falcón et al.2002/2003b<br>Spain (Murcia)	Non-smokers<br>Smokers<br>Residents in rural area<br>Residents in urban area	61<br>25<br>33<br>53	5.8 ± 2.8<br>8.7 ± 4.7<br>5.6 ± 2.6<br>7.3 ± 4.1			Significantly higher levels in smokers and urban area residents. Correlation with number of cigarettes (r:0.42). Negative correlation with Pb concentration and gestational age
Osada et al.2002<br>Japan (Chiba)	Neonates with intrauterine growth restriction<br>Neonates with appropriate growth	21<br>30	13.5 ± 5.6<br>12.0 ± 5.6			No statistically significant differences. Dry/wet ratio: 6.9 +- 1.3
Zhang et al.2004<br>China (Hubei)	High soil pollution. Long-term residents (> 15 years). Single birth	44	33.3 (391.7) [c]	24.2	13.7 - 661.7	Correlation with maternal and cord blood (r:0.89. r:0.30). No association with pre-term labor (gestational age < 37 weeks) or Apgar Score ≤ 7
Ronco et al.2005a,b<br>Chile (Santiago)	Never smokers<br>Smokers (≥ 5 cigarettes/day)	20<br>20	3.3 ± 1.7<br>10.0 ± 3.3			Statistically significant differences. Higher concentrations in maternal side of placenta in smoking mothers. Negative correlation with birth weight in smokers (r:-0.8)
Kutlu et al.2006<br>Turkey	Never smokers<br>Passive smokers: 5 cigarettes<br>Passive smokers: 10 cigarettes<br>Passive smokers: 15 cigarettes<br>Passive smokers: 20 cigarettes<br>Smokers: 5 cigarettes<br>Smokers: 10 cigarettes<br>Smokers: 15 cigarettes<br>Smokers: 20 cigarettes<br>Smokers: 25 cigarettes	30<br>20<br>18<br>17<br>15<br>20<br>20<br>20<br>20<br>10	0.3 ± 0.1<br>1.4 ± 1.6<br>2.6 ± 0.8<br>3.9 ± 0.7<br>4.1 ± 0.8<br>4.5 ± 1.0<br>7.0 ± 1.2<br>12.0 ± 0.7<br>18.5 ± 2.9<br>23.1 ± 3.0			Higher levels with higher tobacco exposure. Positive correlation with Pb in smokers (r:0.999) and non-smokers (r:0.987)
Sorkun et al.2007<br>Turkey (Denizli)	Never smokers, rural area<br>Never smokers, industrial area<br>Smokers (pregnancy or 12 months)	30<br>29<br>33	6.3 ± 2.0<br>8.0 ± 2.3<br>10.5 ± 3.7			Statistically significant differences. Morphological changes and Zn concentration levels in smokers and those from industrial areas
Klapec et al.2008<br>Croatia (Osijek)	Neonates with intrauterine growth restriction<br>Neonates with appropriate growth	49<br>36	10.2 ± 5.2<br>8.0 ± 4.1	9.1<br>8.0	2.6 - 27.0<br>2.1 - 19.2	Statistically significant differences. No association with birth weight. Placental selenium predicts birth weight for full-term neonates with appropriate growth

Supplemental Material, Table S3 (cont) . Studies on Cadmium (Cd) levels in placenta (1976-2011): main results (in ng/g wet weight) in chronological order

Reference, year country	Group characteristics	n	Arithmetic mean ± SD	Median	Rank	Main results
Terrones et al.2008<br>Mexico (Aguascalientes)	Pregnancies with oligoamnios<br>Normal pregnancies	20<br>20	8.9 ± 1.2<br>4.1 ± 0.8			Statistically significant difference
Llanos et al.2009<br>Chile (Santiago)	Term neonates with low birth weight<br>Term neonates with normal weight	20<br>20	8.3 ± 1.0<br>3.8 ± 0.3			Statistically significant differences. Higher levels did not correlate with changes in oxidative stress parameters and/or anti-oxidative enzyme activity
Stasenko et al.2009<br>Croatia (Zagreb)	Non-smokers (> 1 year)<br>Smokers (≤ 20 cigarettes/day)	109<br>99	10.3 ± 4.3<br>22.1 ± 6.8	9.7<br>21.4		Statistically significant differences. Correlation with birth length (r:-0.184), weight (r:-0.176). Cd could have a specific role in endocrine disruption of leptin synthesis
Al-Saleh et al.2010<br>Saudi Arabia (Al-Kharj)	Women hospitalized for delivery; residents for a minimum of one year	1578	7.5 ± 19.3	5.8	0.0 - 727.2	Cd levels in maternal and placental tissues were correlated (r:0.106, p=0). Cd predictors were mother's age, maternal Cd levels, placental Pb levels and placental Hg levels, with very low B-coefficients (0.103, 0.069, 0.074 and –0.069, respectively)
Guo et al. 2010<br>China (Guiyu, Chaonan)	Mothers from Guiyu, e-waste recycling area<br>Controls from Chaonan (20 km southwest)	101<br>119		108.7<br>104.1	0.4 - 415.4<br>2.3 - 393.5	Negative correlation with maternal educational level and distance from home to street. Positive correlation with cooking time during pregnancy and housing ventilation. No correlation with birth length, weight or gestational age
Kippler et al.2010<br>Bangladesh (Matlab)	Non-smokers, rural area	44	21.7	18.3	6.7 - 82.0	Differences between different samples from the same placenta, no centre-peripheral gradient. Correlation with urinary maternal Cd (r:0.5). Dry/wet ratio: 5.7
Needham et al.2011<br>Denmark (Faroe I.)	Normal births in a fishing community	15		35.0		Cd showed a 100-fold excess in placenta versus cord blood. Ratio to cord blood: 107. Poor correlation with cord blood (r:0.12)
Tekin et al.2011a<br>Turkey (Ankara)	Homozygote genotype for metallothionein 2A<br>Heterozygote for MT2A polymorphism	83<br>12	3.5 ± 3.3<br>1.4 ± 1.1	2.2<br>0.8	0.3 - 13.8<br>0.5 - 3.8	Statistically significant differences. Only a small amount of Cd was transferred to fetus. No correlation with birth weight, length or head circumference

[a] **Full text was unavailable (data incorporated into the table are obtained from the abstracts or quoted in other papers)**
[b] **Original values expressed in micrograms/g. probably erroneously (Iyengar and Rapp 2001b)**
[c] **Geometric mean**

Supplemental Material, Table S4. Studies on Lead (Pb) levels in placenta (1976-2011): main results (in ng/g wet weight) in chronological order

Reference, year country	Group characteristics	n	Arithmetic mean ± SD	Median	Rank	Main results
Fahim et al.1976<br>USA (Missouri)	Rolla, lead mining area	253	70.0 ± 0.3			No statistically significant differences. Higher levels in full-term deliveries and early membrane rupture. Higher concentration of Pb in membranes than in placenta
	Augusta	249	60.0 ± 0.1			
Karp et al.1977<br>USA (Southeast)	Total	58	293.0 ± 25.0			Difference between cities not statistically significant. Pb level correlated negatively with enzyme activity of steroid sulfatase. This would suggest an inhibitory effect of the metal on enzyme activity
	Augusta	19	275.0 ± 50.0			
	Birmingham	22	296.0 ± 34.0			
	Charlotte	17	312.0 ± 50.0			
Wibberley et al.1977<br>UK (Birmingham)	Indian women, normal neonates	21	188.3 ± 113.3		78.3 - 593.3	Placentae of 7% of normal neonates and 61% of stillbirths and neonatal deaths had Pb levels >1.5 µg/g. Pb could accumulate in times of fetal stress. Higher levels in winter for European women and in spring for Indian women
	European women, normal neonates	24	155.0 ± 106.7		25.0 - 441.7	
	European, neonatal deaths or malformations	13	248.3 ± 115.0		88.3 - 445.0	
	European, neonatal deaths	14	288.3 ± 95.0		120.0 - 461.7	
	European, stillbirths with no malformations	9	241.7 ± 83.3		101.7 - 365.0	
	Indian and European, normal brothers	21	125.0 ± 31.7			
	European, low neonatal birth (< 2.500 g)	24	160.0 ± 46.7		35.0 - 265.0	
Hubermont et al.1978<br>Belgium (Libramont)	Deliveries in a rural area	70	111.0	89.0	43.0 - 280.0	Levels higher than cord blood and lower than maternal blood. Positive correlation between Pb in drinking water and placental level. Statistically significant differences
	Pb in drinking water < 50 µg/l	41	97.0	82.0	44.0 - 296.0	
	Pb in drinking water > 50 µg/l	29	133.0	120.0	71.0 - 280.0	
Roels et al.1978<br>Belgium	European (Afro-Asians excluded)	474	83.8 ± 51.0	74.7	11.0 - 395.0	Placenta would not seem to be an efficient barrier for Pb. Positive correlation with maternal (r:0.22) and cord blood levels (r:0.28). No statistically significant differences by area of residence or tobacco use. No relation with maternal age, paternal occupation, drinking habits, gestational age, parity and birth weight
	Non-smokers	333	83.0 ± 851.0	73.3	11.0 - 395.0	
	Smokers (> 3 cigarettes/day)	109	84.9 ± 44.0	79.0	12.0 - 276.0	
	Rural and semirural area	231	82.0 ± 82.0	70.0	11.0 - 388.0	
	Urban and industrial area	236	85.0 ± 85.0	77.0	13.4 - 395.0	
Khera et al.1980<br>UK (Birmingham)	All occupationally exposed (pottery industry)	45	350.0 ± 230.0			Higher levels than in previous study (1971) with fresh placenta: 0.12 µg/g. This difference ascribed to continued storage at –20º C, coupled with the method of sample preparation, which entails the prior removal of excess fluid. Higher concentrations with occupation painters versus others), longer occupational exposure and maternal age. Weak correlation with maternal blood (r:0.16)
	Stillbirths	20	450.0 ± 320.0			
	Fetal distress registered	10	430.0 ± 190.0			
	Malformed live-births	9	320.0 ± 140.0			
	Women unexposed last 2 years	8	290.0 ± 90.0			
Tsuchiya et al.1984<br>Japan (Nagoya)	Urban and industrial environment	110	45.0 ± 34.0		5.0 - 174.0	Positive correlation with cord blood (r:0.402. p<0.01)
Korpela et al.1986<br>Finland	Full-term deliveries; healthy neonates	6	22.6 ± 15.7			Placenta does not accumulate Pb (this accumulates in amniotic membranes). Fetal similar to maternal exposure
Mayer-Popken et al.1986<br>Germany	Female worker suffering lead poisoning	1	200.0			Pregnancy was terminated for medical reasons
Schramel et al.1988<br>Germany (Munich)	Urban environment	33	18.7 ± 7.3			Positive correlation with maternal (r:0.69) and cord blood (0.67). No correlation with maternal milk. Dry/wet ratio: 6.2
Truska et al.1989<br>Czech Republic	Non-smokers, industrial area	50	41.9 ± 26.5	3.5		Pb does not accumulate in placenta. Lower levels than maternal and cord erythrocytes and plasma. Positive correlation with maternal erythrocytes and plasma in semirural sample
	Non-smokers, semirural area	50	43.2 ± 29.7	4.0		
Baghurst et al.1991<br>Australia (Port Pirie)	Normal deliveries and neonates	22	80.0[b]			No statistically significant differences. Lower levels than membranes, maternal and cord blood. Placental lead concentration positively associated with all blood lead determinations, with the correlation being strongest for the average of all antenatal measurements (r:0.32, p=0.003)
	Early membrane rupture (≥ 24 h)	18	68.3[b]			
	Stillbirths	9	126.7[b]			
	Pre-term deliveries (≤ 36 weeks)	23	110.0[b]			
	High Pb level in maternal blood 14-20w	7	106.7[b]			
	High Pb level in cord blood	7	160.0[b]			

Supplemental Material, Table S4(cont) . Studies on Lead (Pb) levels in placenta (1976-2011): main results (in ng/g wet weight) in chronological order

Reference, year country	Group characteristics	n	Arithmetic mean ± SD	Median	Rank	Main results
Loiacono et al.1992[c] Yugoslavia (Titova Mitrovica, Pristina)	Titova Microvica (lead smelter)<br>Pristina (unexposed to lead smelter)	106<br>55	17.7 ± 11.9<br>9.2 ± 3.1			Statistically significant differences. Positive correlation with maternal and cord blood (r:0.506. r:0.515. p<0.0001). No association with birth weight or gestational age
Radomanski et al.1992 [a] Poland		127	60.0			
Fagher et al.1993 Sweden (Lund), Poland (Bialystok)	Pre-term cesarean section<br>Full-term cesarean section	17<br>11	50.0 ± 33.3<br>33.3 ± 33.3			No statistically significant differences. Correlation between maternal blood level and gestational age. Polish women had higher levels in blood
Saxena et al.1994 India (Lucknow)	Normal neonates<br>Abnormal delivery cases	192<br>25	179.8 ± 117.9<br>208.8 ± 125.6			No statistically significant differences. No association with other socioenvironmental factors. Positive correlation with maternal and cord blood (γ:0.33, γ:0.36)
Baranovska 1995 Poland (Silesia)	Heavily polluted area	24	83.3 ± 35.0	95.0	8.3 - 133.3	Placenta does not act as a barrier. High levels in maternal and cord blood
Díaz-Barriga et al.1995[a] Mexico	Agricultural zone<br>Smelting and metallurgy activities area	16<br>21	58.5 ± 8.3<br>122.5 ± 19.9		27.1 - 137.0<br>23.2 - 384.0	
Baranowski et al.1996 Poland (Silesia)	Healthy full-term newborns in residents of a heavily polluted area (Upper Silesia)	28	83.3			Placenta is a better barrier for Cd than for Pb
Centeno et al.1996 USA	Patients soon after delivery	25	5.1 ± 6.3		0.9 - 167.2	
Lagerkvist et al.1996 Sweden	Area exposed to lead smelter<br>Unexposed area	49<br>53	12.4 ± 10.4<br>10.4 ± 6.2			No statistically significant differences. Pb does not accumulate, levels 2-3 fold lower than maternal and cord blood. No uniform distribution. Associated with average maternal blood
Yang et al.1997 China (Shangai)	Occupational exposure (lamp factory)<br>Unexposed controls	9<br>9	4.9 ± 3.3<br>5.5 ± 3.6			No statistically significant differences
Koplov et al.1998 Russia (Arctic)	Norilsk<br>Salekhard	?<br>?	102.4 ± 6.9<br>79.2 ± 14.3			
Reichrtova et al.1998a,b Slovakia (Bratislava, Spisska Nova Ves, Krompachy, Stara Lubovna)	Industrially polluted region, heavy traffic<br>Iron mining region, intermediate traffic<br>Small city, copper mining, light traffic<br>Rural region, no industry, high traffic	50<br>50<br>50<br>50	32.4 ± 37.0<br>12.8 ± 11.7<br>9.4 ± 8.9<br>25.0 ± 21.8	20.4<br>7.1<br>6.0<br>12.8	? - 222.7<br>? - 31.3<br>? - 29.8<br>? - 77.7	Statistically significant differences. Placental Pb concentrations gave a better reflection of traffic than industrial pollution. Transport of Pb particles from basal to chorionic plate
Richter et al.1999[a] Czech Republic			11.3 ± 5.8			
Li et al.2000 [d] China (Shangai)	Non-occupationally exposed	153	1.1 ± 1.1	0.9	0.0 - 6.1	Higher placental levels in mothers with higher levels in blood. Positive correlation between maternal and cord blood (r:0.714, p<0.0001)
Osman et al.2000 Sweden (Solna)	Deliveries in Solna	89	8.7			Women who, prior to their pregnancy, had consumed wine once a week or more had higher placental lead levels
Zadorozhnaja et al.2000 Ukraine (Kyiv, Dniprodzerzhinsk)	Urban area. Industrial pollution	200		<24.0 (LOD)	LOD – 79.0	Lead detected in only 22%

Supplemental Material, Table S4(cont) . Studies on Lead (Pb) levels in placenta (1976-2011): main results (in ng/g wet weight) in chronological order

Reference, year country	Group characteristics	n	Arithmetic mean ± SD	Median	Rank	Main results
Odland et al.2001/2004 Russia (Nikel, Monchegorsk, Arkhangelsk), Norway (Kirkenes, Hammerfest, Bergen)	All women (Arctic and sub-Arctic regions)<br>Russian women<br>Norwegian women	263<br>113<br>150		13.3<br>18.3<br>4.8	5.0 - 95.0<br>5.0 - 95.0<br>5.0 - 88.3	Statistically significant differences between Russian and Norwegian women. Weak correlation with tobacco. Associated with maternal and cord blood levels (p<0.005)
Pereg et al.2001 Canada (Quebec)	Non-smokers<br>Smokers (3 - 25 cigarettes/day)	23<br>17	6.4 ± 0.3<br>7.2 ± 0.3			No statistically significant differences
Piasek et al.2001 Croatia (Zagreb)	Non-smokers (> 1 year)<br>Smokers	24<br>27	48.1 ± 7.8<br>34.4 ± 4.8			No statistically significant differences. No differences between central and peripheral samples of placenta
Falcón et al.2002/2003a Spain (Murcia)	Normal full-term deliveries<br>Early membrane rupture/pre-term deliveries<br>Residents in rural area<br>Residents in urban area<br>Non-smokers<br>Smokers	71<br>18<br>33<br>53<br>61<br>25	17.2 ± 8.3<br>25.7 ± 12.0<br>14.2 ± 5.8<br>21.6 ± 10.4<br>19.2 ± 9.9<br>17.8 ± 9.0	14.1<br>23.4	5.9 - 40.3<br>9.0 - 50.7	Significantly higher levels in adverse neonatal outcomes and urban area residents. Negative correlation with gestational age. No association with smoking
Zagrodzki et al.2003 Poland (Krakow, Bieszczady)	Healthy non-smokers. Full-term deliveries<br>Industrial polluted area (Cracow)<br>Rural area (Bieszczady)	23<br>10<br>13	51.6 ± 18.0<br>55.1 ± 19.1<br>48.9 ± 17.3	49.5<br>46.4<br>49.8	12.0 - 85.1<br>33.2 - 85.1<br>12.0 - 81.4	No statistically significant differences. Positive correlation with maternal age, body weight before and at the end of pregnancy, length of fetal plate
Lafond et al.2004 Canada (Quebec)	Healthy, unexposed women	30	3.4 ± 1.0	1.0		Higher lead levels in maternal blood associated with lower placental calcium levels
Kutlu et al.2006 Turkey	Never smokers<br>Passive smokers: 5 cigarettes<br>Passive smokers: 10 cigarettes<br>Passive smokers: 15 cigarettes<br>Passive smokers: 20 cigarettes<br>Smokers: 5 cigarettes<br>Smokers: 10 cigarettes<br>Smokers: 15 cigarettes<br>Smokers: 20 cigarettes<br>Smokers: 25 cigarettes	30<br>20<br>18<br>17<br>15<br>20<br>20<br>20<br>20<br>10	2.8 ± 0.8<br>11.8 ± 2.2<br>15.9 ± 0.2<br>25.8 ± 6.9<br>29.8 ± 0.9<br>42.2 ± 3.7<br>74.3 ± 12.3<br>139.2 ± 6.8<br>209.1 ± 5.7<br>258.9 ± 2.4			Higher levels with higher smoking exposure. Positive correlation with cadmium in smokers (r:0.999, p<0.01) and non-smokers (r:0.987, p<0.01)
Klapec et al.2008 Croatia (Osijek)	Neonates with intrauterine growth restriction<br>Neonates with appropriate growth	49<br>36	52.8 ± 19.4<br>46.6 ± 18.7	52.4<br>45.2	16.0 - 112.1<br>15.5 - 93.5	No statistically significant differences. No association with birth weight. Placental selenium predicts birth weight for full-term neonates with appropriate growth
Terrones et al.2008 Mexico (Aguascalientes)	Pregnancies with oligoamnios<br>Normal pregnancies	20<br>20	33.8 ± 4.5<br>13.8 ± 2.7			Statistically significant difference
Ahamed et al.2009 India (Lucknow)	Vaginal deliveries. Industrial area<br>Pre-term deliveries<br>Full-term deliveries	60<br>29<br>31	330.0 ± 210.0<br>390.0 ± 200.0<br>270.0 ± 150.0			Statistically significant differences. Lead-induced oxidative stress may be one of the underlying mechanism(s) of pre-term delivery
Llanos et al. 2009 Chile (Santiago)	Term neonates with low birth weight<br>Term neonates with normal weight	20<br>20	35.0 ± 6.7<br>6.7 ± 1.5			Statistically significant differences. Higher levels did not correlate with changes in oxidative stress parameters and/or anti-oxidative enzyme activity
Stasenko et al.2009 Croatia (Zagreb)	Non-smokers (> 1 year)<br>Smokers (≤ 20 cigarettes/day)	109<br>99	20.0 ± 17.7<br>26.3 ± 22.5			Statistically significant differences. Correlation with birth weight (r:-0.164, p=0.018)

Supplemental Material, Table S4(cont) . Studies on Lead (Pb) levels in placenta (1976-2011): main results (in ng/g wet weight) in chronological order

Reference, year country	Group characteristics	n	Arithmetic mean ± SD	Median	Rank	Main results
Al-Saleh et al.2010<br>Saudi Arabia ((Al-Kharj)	Women hospitalized for delivery; residents for a minimum of one year	1576	96.5 ± 362.7	75.0	0.0 - 1300.0	Lead levels in cord blood and placenta were negatively associated (r:-0.063, p=0.014). Levels were inversely associated with the duration of applying henna on hair and hands, time since home was built, number of cups of coffee and tea consumed. Application of skin-lightening creams was associated with higher levels of placental Pb
Gundacker et al. 2010<br>Austria (Vienna)	Healthy mothers in urban environment	31		25.8	10.7 - 75.4	Transfer and placental retention were observed. Women with higher placental lead levels reported miscarriage more often. Maternal poultry and mushroom consumption was inversely related with lead accumulation. Lead was a significant predictor of birth length and weight
Guo et al. 2010<br>China (Guiyu, Chaonan)	Mothers from Guiyu, e-waste recycling area<br>Controls from Chaonan (20 km southwest)	101<br>119		301.4<br>165.8	6.5 - 3465.2<br>4.5 - 3176.1	Statistically significant differences. Positive correlation with the paternal work linked to e-waste, residence in Guiyu and length of residence. No correlation with birth length/weight
Singh et al.2010<br>India (Lucknow)	Healthy women not occupationally exposed<br>Nulliparous<br>Multiparous	60<br>23<br>37	350.0 ± 300.0<br>270.0 ± 280.0<br>400.0 ± 300.0	300.0	0.0 - 1200.0	Placental lead had significant negative correlation with zinc (r:-0.35, p<0.05), and was significantly higher in multiparous women
Needham et al.2011<br>Denmark (Faroe I.)	Normal births in a fishing community	15		53.0		Although poorly correlated, the average lead concentrations were quite similar in cord blood and milk but higher in the placenta. Ratio to cord blood: 8.9, correlation coefficient: 0.34
Tekin et al.2011b<br>Turkey (Ankara)	All (healthy women with healthy babies)<br>Homozygote genotype for metallothionein 2A<br>Heterozygote for MT2A polymorphism	91<br>79<br>12	1.3 ± 0.5<br>1.3 ± 0.4<br>1.6 ± 0.7	1.2<br>1.2<br>1.5	0.6 - 2.9<br>0.6 - 2.5<br>0.8 - 2.9	Statistically significant differences. Placental Pb levels were not correlated with gestational age, birth weight, length and head circunference of the newborns. No significant correlations between cord blood, placental and maternal Pb levels

[a] **Full text was unavailable (data incorporated into the table are obtained from the abstracts or quoted in other papers)**
[b] **Geometric mean**
[c] **The original values are expressed in nmol/g, probably erroneously**
[d] **Units for the same values are expressed in µg/kg in the tables and in µmol/kg in the text of the paper, appearing the latter option most consistent with the data**

SUPPLEMENTAL MATERIAL, REFERENCE LIST

1. Ahamed M, Mehrotra P, Kumar P, Siddiqui M. 2009. Placental lead-induced oxidative stress and preterm delivery. Environmental Toxicology and Pharmacology 27:70-74.
2. Al Saleh I, Shinwari N, Mashhour A, Mohamed GD, Rabah A. 2011. Heavy metals (lead, cadmium and mercury) in maternal, cord blood and placenta of healthy women. Int J Hyg Environ Health 214:79-101.
3. Baghurst PA, Robertson EF, Oldfield RK, King BM, McMichael AJ, Vimpani GV et al. 1991. Lead in the placenta, membranes, and umbilical cord in relation to pregnancy outcome in a lead-smelter community. Environ Health Perspect 90:315-320.
4. Baranowska I. 1995. Lead and cadmium in human placentas and maternal and neonatal blood in a heavily polluted area measured by graphite furnace atomic absorption spectrometry. Occup Environ Med 52:229-232.
5. Baranowski J, Norska-Borówka I. 1996. Determination of lead and cadmium in placenta, umbilical cord blood and maternal blood using pulse differential polarography. Metal ions in Biology and Medicine. Paris, John Libbey Eurotext, 4: 654-656.
6. Berlin M, Blanks R, Catton M, Kazantzis G, Mottet NK, Samiullah Y. 1992. Birth weight of children and cadmium accumulation in placentas of female nickel-cadmium (long-life) battery workers. IARC Sci Publ 257-262.
7. Bush PG, Mayhew TM, Abramovich DR, Aggett PJ, Burke MD, Page KR. 2000. A quantitative study on the effects of maternal smoking on placental morphology and cadmium concentration. Placenta 21:247-256.
8. Capelli R, Minganti V, Semino G, Bertarini W. 1986. The presence of mercury (total and organic) and selenium in human placentae. Sci Total Environ 48:69-79.
9. Centeno JA, Pestaner JP, Nieves S, Ramos M, Mullick FG, Kaler SG. 1996. The assessment of trace element and toxic metal levels in human placental issues. Metal Ions in Biology and Medicine. Collery P, Corbella J, Domingo JL, Etienne JC, & Uobel JM eds, 4: 522-524.
10. Diaz-Barriga F, Carrizeles L, Calderon J, Batres L, Yanez L, Tabor MW et al. 1995. Measurement of placental levels of arsenic, lead and cadmium as a biomarker of exposure to mixtures. In: Biomonitors and Biomarkers as Indicators of Environmental Change: A Handbook (Butterworth F, Corkum L, and Guzmán-Rincón J eds). New York:Plenum Press, 139-148.
11. Fagher U, Laudanski T, Schutz A, Sipowicz M, Akerlund M. 1993. The relationship between cadmium and lead burdens and preterm labor. Int J Gynaecol Obstet 40:109-114.
12. Fahim MS, Fahim Z, Hall DG. 1976. Effects of subtoxic lead levels on pregnant women in the state of Missouri. Res Commun Chem Pathol Pharmacol 13:309-331.
13. Falcon M, Vinas P, Luna A. 2003a. Placental lead and outcome of pregnancy. Toxicology 185:59-66.
14. Falcon M, Vinas P, Osuna E, Luna A. 2002. Environmental exposures to lead and cadmium measured in human placenta. Arch Environ Health 57:598-602.
15. Falcon M, Vinas P, Perez-Carceles MD, Luna A. 2003b. Placental cadmium and lipid peroxidation in smoking women related to newborn anthropometric measurements. Arch Environ Contam Toxicol 45:278-282.
16. Fiala J, Hruba D, Rezl P. 1998. Cadmium and zinc concentrations in human placentas. Cent Eur J Public Health 6:241-248.
17. Frery N, Nessmann C, Girard F, Lafond J, Moreau T, Blot P et al. 1993. Environmental exposure to cadmium and human birthweight. Toxicology 79:109-118.
18. Galster WA. 1976. Mercury in Alaskan Eskimo mothers and infants. Environ Health Perspect 15:135-140.
19. Grant C, Lalor G, Fletcher H, Potter T, Vutchkov M, Reid M. 2010. Elements in human placentae in Jamaica. West Indian Med J 59:479-485.
20. Gundacker C, Frohlich S, Graf-Rohrmeister K, Eibenberger B, Jessenig V, Gicic D et al. 2010. Perinatal lead and mercury exposure in Austria. Sci Total Environ 408:5744-5749.
21. Guo Y, Huo X, Li Y, Wu K, Liu J, Huang J et al. 2010. Monitoring of lead, cadmium, chromium and nickel in placenta from an e-waste recycling town in China. Sci Total Environ 408:3113-3117.
22. Horvat M, Stegnar P, Byrne AR, Dermelj M. 1988. A study of trace elements in human placenta, blood and hair from the Yugoslav Central Adriatic. In: Trace element analytical chemistry in medicine and biology (Berlin, New York:Walter de Gruyter & Co, 243-250.
23. Hsu CS, Liu PL, Chien LC, Chou SY, Han BC. 2007. Mercury concentration and fish consumption in Taiwanese pregnant women. BJOG 114:81-85.
24. Iyengar GV, Rapp A. 2001. Human placenta as a 'dual' biomarker for monitoring fetal and maternal environment with special reference to potentially toxic trace elements. Part 3: toxic trace elements in placenta and placenta as a biomarker for these elements. Sci Total Environ 280:221-238.
25. Hubermont G, Buchet JP, Roels H, Lauwerys R. 1978. Placental transfer of lead, mercury and cadmium in women living in a rural area. Importance of drinking water in lead exposure. Int Arch Occup Environ Health 41:117-124.
26. Kantola M, Purkunen R, Kroger P, Tooming A, Juravskaja J, Pasanen M et al. 2000. Accumulation of cadmium, zinc, and copper in maternal blood and developmental placental tissue: differences between Finland, Estonia, and St. Petersburg. Environ Res 83:54-66.
27. Karp WB, Robertson AF. 1977. Correlation of human placental enzymatic activity with trace metal concentration in placentas from thres geographical locations. Environ Res 13:470-477.
28. Khera AK, Wibberley DG, Dathan JG. 1980. Placental and stillbirth tissue lead concentrations in occupationally exposed women. Br J Ind Med 37:394-396.
29. Kippler M, Hoque AM, Raqib R, Ohrvik H, Ekstrom EC, Vahter M. 2010. Accumulation of cadmium in human placenta interacts with the transport of micronutrients to the fetus. Toxicol Lett 192:162-168.
30. Klapec T, Cavar S, Kasac Z, Rucevic S, Popinjac A. 2008. Selenium in placenta predicts birth weight in normal but not intrauterine growth restriction pregnancy. J Trace Elem Med Biol 22:54-58.

31. Klopov VP. 1998. Levels of heavy metals in women residing in the Russian Arctic. Int J Circumpolar Health 57 Suppl 1:582-585.
32. Korpela H, Loueniva R, Yrjanheikki E, Kauppila A. 1986. Lead and cadmium concentrations in maternal and umbilical cord blood, amniotic fluid, placenta, and amniotic membranes. Am J Obstet Gynecol 155:1086-1089.
33. Kuhnert BR, Kuhnert PM, Zarlingo TJ. 1988. Associations between placental cadmium and zinc and age and parity in pregnant women who smoke. Obstet Gynecol 71:67-70.
34. Kuhnert PM, Kuhnert BR, Bottoms SF, Erhard P. 1982. Cadmium levels in maternal blood, fetal cord blood, and placental tissues of pregnant women who smoke. Am J Obstet Gynecol 142:1021-1025.
35. Kutlu T, Karagozler AA, Gozukara EM. 2006. Relationship among placental cadmium, lead, zinc, and copper levels in smoking pregnant women. Biol Trace Elem Res 114:7-17.
36. Lafond J, Hamel A, Takser L, Vaillancourt C, Mergler D. 2004. Low environmental contamination by lead in pregnant women: effect on calcium transfer in human placental syncytiotrophoblasts. J Toxicol Environ Health A 67:1069-1079.
37. Lagerkvist BI, Sandberg S, Frech W, Jin T, Nordberg GF. 1996. Is placenta a good indicator of cadmium and lead exposure? Arch Environ Health 51:389-394.
38. Li PJ, Sheng YZ, Wang QY, Gu LY, Wang YL. 2000. Transfer of lead via placenta and breast milk in human. Biomed Environ Sci 13:85-89.
39. Llanos MN, Ronco AM. 2009. Fetal growth restriction is related to placental levels of cadmium, lead and arsenic but not with antioxidant activities. Reprod Toxicol 27:88-92.
40. Loiacono NJ, Graziano JH, Kline JK, Popovac D, Ahmedi X, Gashi E et al. 1992. Placental cadmium and birthweight in women living near a lead smelter. Arch Environ Health 47:250-255.
41. Marques RC, Garrofe DJ, Rodrigues BW, de Freitas RM, de Freitas FM, Malm O. 2007. Maternal mercury exposure and neuro-motor development in breastfed infants from Porto Velho (Amazon), Brazil. Int J Hyg Environ Health 210:51-60.
42. Mayer-Popken O, Denkhaus W, Konietzko H. 1986. Lead content of fetal tissues after maternal intoxication. Arch Toxicol 58:203-204.
43. Moberg WA, Wing K, Tholin K, Sjostrom R, Sandstrom B, Hallmans G. 1992. The relation of the accumulation of cadmium in human placenta to the intake of high-fibre grains and maternal iron status. Eur J Clin Nutr 46:585-595.
44. Needham LL, Grandjean P, Heinzow B, Jorgensen PJ, Nielsen F, Patterson DG, Jr. et al. 2011. Partition of environmental chemicals between maternal and fetal blood and tissues. Environ Sci Technol 45:1121-1126.
45. Odland JO, Nieboer E, Romanova N, Thomassen Y, Hofoss D, Lund E. 2001. Factor analysis of essential and toxic elements in human placentas from deliveries in arctic and subarctic areas of Russia and Norway. J Environ Monit 3:177-184.
46. Odland JO, Nieboer E, Romanova N, Thomassen Y. 2004. Elements in placenta and pregnancy outcome in arctic and subarctic areas. Int J Circumpolar Health 63:169-187.
47. Osada H, Watanabe Y, Nishimura Y, Yukawa M, Seki K, Sekiya S. 2002. Profile of trace element concentrations in the feto-placental unit in relation to fetal growth. Acta Obstet Gynecol Scand 81:931-937.
48. Osman K, Akesson A, Berglund M, Bremme K, Schutz A, Ask K et al. 2000. Toxic and essential elements in placentas of Swedish women. Clin Biochem 33:131-138.
49. Peereboom-Stegeman JH, van der Velde WJ, Dessing JW. 1983. Influence of cadmium on placental structure. Ecotoxicol Environ Saf 7:79-86.
50. Pereg D, Lagueux J, Dewailly E, Poirier G, Ayotte P. 2001. Cigarette smoking during pregnancy: comparison of biomarkers for inclusion in epidemiological studies. Biomarkers 6:161-173.
51. Piasek M, Blanusa M, Kostial K, Laskey JW. 2001. Placental cadmium and progesterone concentrations in cigarette smokers. Reprod Toxicol 15:673-681.
52. Pitkin RM, Bahns JA, Filer LJ, Jr., Reynolds WA. 1976. Mercury in human maternal and cord blood, placenta, and milk. Proc Soc Exp Biol Med 151:565-567.
53. Radomanski Jr, Sikorski R. 1992. Human placenta magnesium adn the contamination with cadmium and lead of placenta. J Perinat Med 20:181.
54. Reichrtova E, Dorociak F, Palkovicova L. 1998b. Sites of lead and nickel accumulation in the placental tissue. Hum Exp Toxicol 17:176-181.
55. Reichrtova E, Ursinyova M, Palkovicova L, Wsolova L. 1998a. Contents and localization of heavy metals in human placentae. Fresenius J Anal chem 361:362-364.
56. Richter J, Hajek Z, Pfeifer I, Subrt P. 1999. Relation between concentration of lead, zinc and lysozyme in placentas of women with intrauterine foetal growth retardation. Cent Eur J Public Health 7:40-42.
57. Roels H, Hubermont G, Buchet JP, Lauwerys R. 1978. Placental transfer of lead, mercury, cadmium, and carbon monoxide in women. III. Factors influencing the accumulation of heavy metals in the placenta and the relationship between metal concentration in the placenta and in maternal and cord blood. Environ Res 16:236-247.
58. Ronco AM, Arguello G, Suazo M, Llanos MN. 2005b. Increased levels of metallothionein in placenta of smokers. Toxicology 208:133-139.
59. Ronco AM, Garrido F, Llanos MN. 2006. Smoking specifically induces metallothionein-2 isoform in human placenta at term. Toxicology 223:46-53.
60. Saxena DK, Singh C, Murthy RC, Mathur N, Chandra SV. 1994. Blood and placental lead levels in an Indian city: a preliminary report. Arch Environ Health 49:106-110.
61. Scaal M, Schweinsberg F, Kaiserling E. 1998. Mercury concentrations in fetuses with malformations. Zentralbl Hyg Umweltmed 201:413-421.
62. Schramel P, Hasse S, Ovcar-Pavlu J. 1988. Selenium, cadmium, lead, and mercury concentrations in human breast milk, in placenta, maternal blood, and the blood of the newborn. Biol Trace Elem Res 15:111-124.
63. Singh J, Singh V, Anand M, Kumar P, Siddiqui M. 2010. Placental Lead and its Interaction with Some Essential Metals among Women from Lucknow, India. Asian Journal of Medical Sciences 32-36.
64. Soria ML, Sanz P, Martinez D, Lopez-Artiguez M, Garrido R, Grilo A et al. 1992. Total mercury and methylmercury in hair, maternal and umbilical

blood, and placenta from women in the Seville area. Bull Environ Contam Toxicol 48:494-501.

65. Sorkun HC, Bir F, Akbulut M, Divrikli U, Erken G, Demirhan H et al. 2007. The effects of air pollution and smoking on placental cadmium, zinc concentration and metallothionein expression. Toxicology 238:15-22.
66. Stasenko S, Bradford EM, Piasek M, Henson MC, Varnai VM, Jurasovic J et al. 2010. Metals in human placenta: focus on the effects of cadmium on steroid hormones and leptin. J Appl Toxicol 30:242-253.
67. Suzuki T, Yonemoto J, Satoh H, Naganuma A, Imura N, Kigawa T. 1984. Normal organic and inorganic mercury levels in the human feto-placental system. J Appl Toxicol 4:249-252.
68. Tabacova S, Baird DD, Balabaeva L, Lolova D, Petrov I. 1994. Placental arsenic and cadmium in relation to lipid peroxides and glutathione levels in maternal-infant pairs from a copper smelter area. Placenta 15:873-881.
69. Tekin D, Kayaalti Z, Aliyev V, Soylemezoglu T. 2011a. The effects of metallothionein 2A polymorphism on placental cadmium accumulation: is metallothionein a modifiying factor in transfer of micronutrients to the fetus? J Appl Toxicol ;doi: 10.1002/jat.1661 [Online 15 April 2011].
70. Tekin D, Kayaalti Z, Soylemezoglu T. 2011b. The effects of metallothionein 2A polymorphism on lead metabolism: are pregnant women with a heterozygote genotype for metallothionein 2A polymorphism and their newborns at risk of having higher blood lead levels? Int Arch Occup Environ Health;[Online 18 Oct 2011].
71. Terrones-Saldivar M, Serrano Díaz L, Avelar Gonzalez F, Rosas Cabral A, Yamamoto Flores L, Reyes Robles M. 2008. Estudio comparativo entre las concentraciones de cadmio y de plomo en placentas de embarazos normales y placentas de embarazos con oligoamnios idiopáticopoi[In Spanish]. Investigación y Ciencia de la Universidad de Aguascalientes 42:11-17
72. Truska P, Rosival L, Balazova G, Hinst J, Rippel A, Palusova O et al. 1989. Blood and placental concentrations of cadmium, lead, and mercury in mothers and their newborns. J Hyg Epidemiol Microbiol Immunol 33:141-147.
73. Tsuchiya H, Mitani K, Kodama K, Nakata T. 1984. Placental transfer of heavy metals in normal pregnant Japanese women. Arch Environ Health 39:11-17.
74. van Hattum B, de Voogt P, Copius Peereboom JW. 1981. An analytical procedure for the determination of cadmium in human placentae. Int J Environ Anal Chem 10:121-133.
75. Ward NI, Watson R, Bryce-Smith D. 1987. Placental element levels in relation to fetal development for obstetrically 'normal' births. A study of 37 elements. Evidence for effects of cadmium, lead and zinc on fetal growth, and for smoking as a source of cadmium. Int J Biosoc Res 9:63-81.
76. Wibberley DG, Khera AK, Edwards JH, Rushton DI. 1977. Lead levels in human placentae from normal and malformed births. J Med Genet 14:339-345.
77. Yang J, Jiang Z, Wang Y, Qureshi IA, Wu XD. 1997. Maternal-fetal transfer of metallic mercury via the placenta and milk. Ann Clin Lab Sci 27:135-141.
78. Zadorozhnaja TD, Little RE, Miller RK, Mendel NA, Taylor RJ, Presley BJ et al. 2000. Concentrations of arsenic, cadmium, copper, lead, mercury, and zinc in human placentas from two cities in Ukraine. J Toxicol Environ Health A 61:255-263.
79. Zagrodzki P, Zamorska L, Borowski P. 2003. Metal (Cu, Zn, Fe, Pb) concentrations in human placentas. Cent Eur J Public Health 11:187-191.
80. Zhang YL, Zhao YC, Wang JX, Zhu HD, Liu QF, Fan YG et al. 2004. Effect of environmental exposure to cadmium on pregnancy outcome and fetal growth: a study on healthy pregnant women in China. J Environ Sci Health A Tox Hazard Subst Environ Eng 39:2507-2515.
